# Reliability issues of lead-free solder joints in electronic devices

**DOI:** 10.1080/14686996.2019.1640072

**Published:** 2019-07-11

**Authors:** Nan Jiang, Liang Zhang, Zhi-Quan Liu, Lei Sun, Wei-Min Long, Peng He, Ming-Yue Xiong, Meng Zhao

**Affiliations:** a School of Mechatronic Engineering, Jiangsu Normal University, Xuzhou, China; b State Key Laboratory of Advanced Welding and Joining, Harbin Institute of Technology, Harbin, China; c Institute of Metal Research, Chinese Academy of Sciences, Shenyang, China; d National Key Laboratory of Science and Technology on Helicopter Transmission, Nanjing University of Aeronautics and Astronautics, Nanjing, China; e State Key Laboratory of Advanced Brazing Filler Metals and Technology, Zhengzhou Research Institute of Mechanical Engineering, Zhengzhou, China

**Keywords:** Lead-free solder, reliability, IMC, crack, failure, 40 Optical, magnetic and electronic device materials, 106 Metallic materials, 103 Composites, 201 Electronics / Semiconductor / TCOs, 212 Surface and interfaces, 503 TEM, STEM, SEM

## Abstract

Electronic products are evolving towards miniaturization, high integration, and multi-function, which undoubtedly puts forward higher requirements for the reliability of solder joints in electronic packaging. Approximately 70% of failure in electronic devices originates during the packaging process, mostly due to the failure of solder joints. With the improvement of environmental protection awareness, lead-free solder joints have become a hot issue in recent years. This paper reviews the research progress on the reliability of lead-free solder joints and discusses the influence of temperature, vibration, tin whisker and electromigration on the reliability of solder joints. In addition, the measures to improve the reliability of solder joints are analyzed according to the problems of solder joints themselves, which provides a further theoretical basis for the study of the reliability of solder joints of electronic products in service.

## Graphical Abstract

**Figure:**
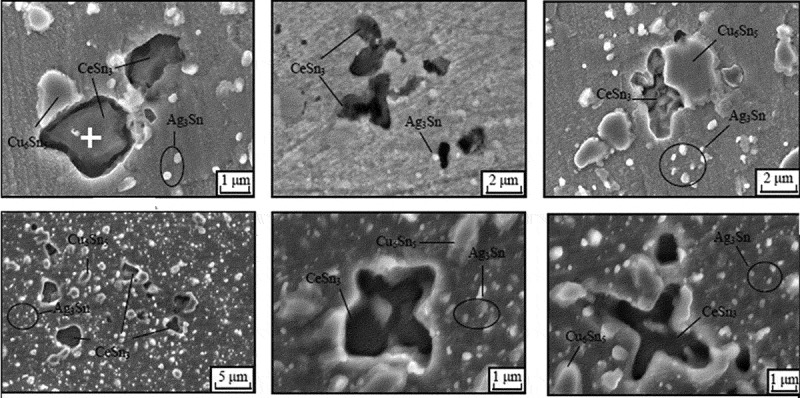
Reproduced by permission from Zhang et al., J Mech Eng. 2012;48(24):56–60.

## 1. Introduction

With the rapid development of the electronics industry, electronic products are gradually turning to miniaturization and multi-functionality. In order to meet people’s needs, electronic packaging technology is developing in the direction of high density, high functionality, and high integration. Electronic packaging technology is the technical basis of system packaging technology, providing electronic and mechanical connections and protection for electronic chips [1]. Therefore, electronic packaging has been widely used in the electronics industry and has occupied an important position.

For a long time, tin-lead alloys have been the main welding materials in the field of electronic packaging because of their good electrical conductivity, mechanical properties and low cost [2]. With the rapid development of electronic packaging technology, the thermal and mechanical loads that solder joints need to withstand exceed the maximum allowable, and the performance requirements for brazing are gradually increasing. Traditional tin-lead solder can not meet the requirements of the electronics industry and require the development of high-performance lead-free solder materials [3]. Especially on 13 February 2003, the EU issued the Waste Electrical and Electronic Equipment (WEEE) and Restriction of Hazardous Substances (RoHS) directives in the form of official gazettes [4], which accelerated the lead-free process. In the process of lead-free transition, the reliability of lead-free solder joints is one of the most prominent problems caused by packaging materials and process changes. The reliability of the package is a significant part of the reliability of the electronic product. The reliability of the electronic package is evaluated by evaluating the ability of the package system to resist the degradation of the function of the electronic device [5]. In the structure of the electronic chip package, the solder joint can not only provide the electrical connection between the substrate and the chip but also be the heat dissipation channel between the chip and the substrate [6]. Solder joints are usually the weakest part of an electronic product and the most likely to fail. Therefore, the reliability of solder joints in electronic packaging affects the reliability of electronic products and even the whole electronic system. There are many external factors that lead to solder joint failure, such as temperature [7], vibration [8], tin whisker [9] and electromigration [10]. It is critical to study solder joint reliability during service in these specific environments, so as to judge the life of electronic products.

This paper reviews the research progress on solder joint reliability of lead-free solder joints, discusses the impact of temperature, vibration, tin whisker and electromigration on solder joint reliability, and provides a further theoretical basis for the study of solder joint reliability of electronic products in service.

## 2. Influence of temperature on lead-free solder joints

The melting temperature of lead-free solder is relatively low, so the variation of temperature load has a significant effect on the properties of the solder joint [11]. Currently, thermal protection is used to maintain the ambient temperature of the electronic device to ensure that the electronic product can operate reliably under extreme conditions. Temperature can change the microstructure and internal stress of the solder joint, which is one of the main factors affecting the solder joint failure. Solder joint failure is the primary cause of electronic component failure, so the influence of temperature on solder joint reliability needs to be further studied. The effect of temperature on solder joint reliability mainly includes aging, thermal cycling, and thermal shock.

### 2.1. Aging

When the molten solder contacts the substrate, an intermetallic compound (IMC) is formed at the interface between the solder and the base material. In the subsequent aging process, the microstructure of the solder joint will be coarsened, the interface IMC will continue to grow, and the stress concentration caused by it will promote the initiation and expansion of the solder joint crack. As the interface IMC is a brittle phase, when the interface IMC reaches a certain thickness it will significantly reduce the mechanical properties of the solder joints, and eventually cause the failure of the solder joints and reduce the reliability of the solder joints [12]. Therefore, it is extremely important to study the effect of aging on the reliability of solder joints.

**Figure 1.**
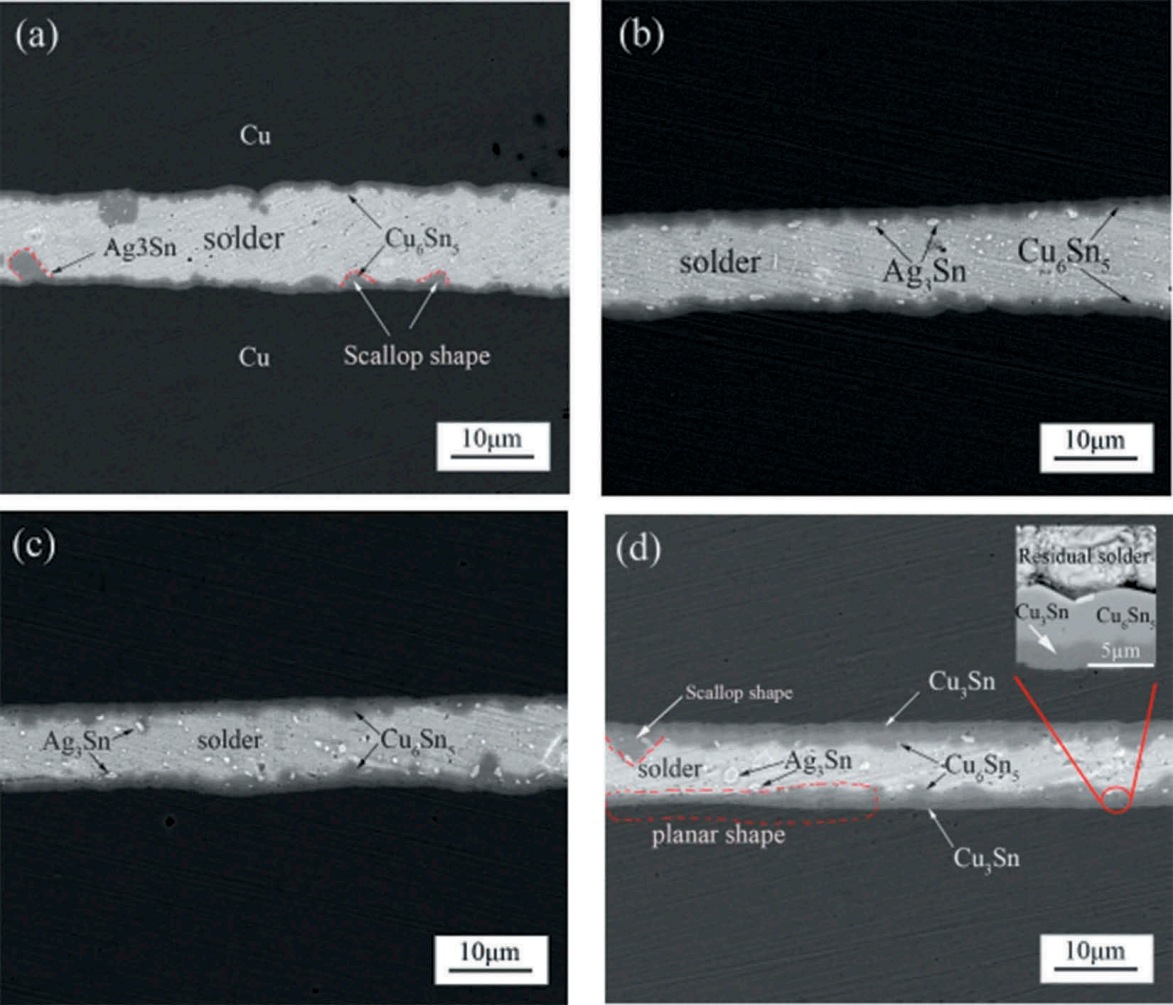
Cross-sectional SEM images of Cu/Sn3.0Ag0.5Cu/Cu solder at different aging times of (a) 0 h, (b) 120 h, (c) 240 h and (d) 360 h [14], reproduced by permission from Li et al., Materials. 2018;11(1):84–95.

With the extension of aging time, the interface layer of the solder joint is continuously thickened, the strength of the solder joint is gradually reduced, and its reliability will be significantly reduced. Gancarz et al. [13] studied the effect of aging on the reliability of SnZnNd lead-free solder joints by electron microscopy and STR-1000 micro-joint strength tester. It is found that the tensile force of the solder joint gradually decreased with the increasing of the aging time. Because with the increase of aging time, a large number of Nd_3_Sn intermetallic compounds are formed at the interface, and these IMCs are brittle phases, which can easily lead to stress concentration under the action of applied load, thus leading to the initiation and expansion of solder joint cracks, and ultimately the failure of electronic components. Li et al. [14] studied the growth of interface IMC in the aging process. Figure 1 shows cross-sectional scanning electron microscopy (SEM) images of Cu/Sn3.0Ag0.5Cu/Cu solder joints at different aging times. It can be seen that the thickness of Cu_6_Sn_5_ IMC increases significantly with the increase of aging time. The Ag_3_Sn particles are dispersed in the solder joints, as shown in Figure 1(a). Ag_3_Sn is a relatively stable compound that can play a role in increasing solder joint strength and improving solder joint fatigue life [15]. The Ag_3_Sn particle size inside the solder joint increases significantly after aging, as can be seen in Figure 1(b–d). This will cause a change in the direction of rotation in the high-energy release zone, resulting in a gradual shift in the fracture mechanism of the solder joint, thereby reducing the reliability of the solder joint. The Cu_3_Sn phase is formed between Cu_6_Sn_5_ layer and Cu substrate, as shown in Figure 1(d). Mustafa et al. [16] studied the effect of aging on the fatigue life of solder joints and found that the fatigue life of solder joints gradually decreased with the extension of aging time. Since in the aging process, the size of intermetallic compounds and grains gradually increases, which is prone to stress concentration, resulting in grain coarsening and recrystallization in the high-strain area, crack initiation along the grain boundary intersection and weak grain boundary, thus significantly reducing the fatigue life of solder joints. Wang et al. [17] studied the effect of isothermal aging on the reliability of Sn3.0Ag0.5Cu solder joints. It was found that the tensile strength and shear strength of solder joints decreased as the aging time increased. The reason is that with the increase of aging time, the atomic diffusion rate increases and the internal structure of the solder matrix coarsens obviously, resulting in the decrease of solder joint strength. However, the tensile strength of the solder joints is slightly improved at the initial stage of aging. Since the interface IMC is very thin at the initial stage of aging, and the germination and growth of the interface IMC are suppressed due to the restraining action of the brazing material and the base metal, resulting in an increase in the tensile strength of the solder joint [18].

**Figure 2.**
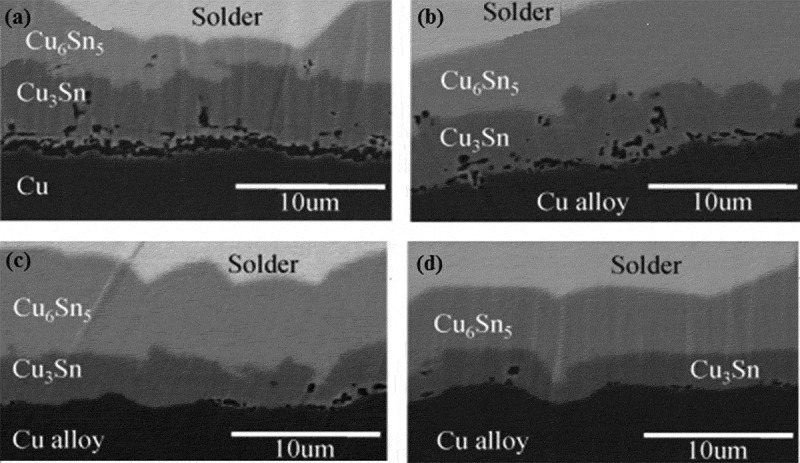
SEM images of Cu-xNi/Sn0.7Cu solder following annealing at 423 K for 2000 h: (a) x = 0, (b) x = 0.09, (c) x = 0.2 and (d) x = 0.4% [23], reproduced by permission from Maeshima et al., Mater Des. 2016;103:106–113.

Cu has good solderability and thermal conductivity and has been widely used as a substrate material in electronic packaging [19]. At present, Ni, Ag, and Au were plated on Cu substrate to inhibit the growth of interfacial IMC. Ni is used as a barrier between solder and Cu substrate. Since the reaction rate between solder and Ni is effectively lower than that between Cu and solder, thereby causing the thickness of interfacial IMC is lower [20]. On Ni/Ag and Ni/Au plated substrates, Au and Ag can inhibit the velocity that Ni and Sn reactions to form Ni_3_Sn_4,_ so its IMC layer is relatively thinner than that of Ni plated substrates [21]. The electroless nickel/immersion gold layer has also been extensively used because of its superior wettability. The Au layer can prevent the surface of Cu substrate from being corroded and oxidized. The electroless Ni plating layer can significantly reduce the interdiffusion rate of Cu atoms and Sn atoms [22]. The addition of trace alloying elements to the Cu substrate can change the microstructure of the substrate, thus improving the performance of the substrate. Maeshima et al. [23] discussed the effect of adding Ni on Cu substrates on the IMC of solder and found that the addition of Ni can effectively inhibit the germination and growth of IMC. The main reason is that the addition of Ni element causes the IMC to form a layer (Cu_1-x_Ni_x_)_6_Sn_5_ near the side of the Cu substrate, thereby inhibiting the germination and growth of the IMC during the aging process and improving the reliability of the solder joint. Figure 2 shows the interfacial structure of Ni content after adding different contents of Ni to Sn-0.7Cu. It can be seen that the thickness of Cu_3_Sn layer decreases gradually with the increase of Ni content, and the thickness of Cu_6_Sn_5_ layer increases remarkably. The higher the activation energy, the faster the growth rate of IMC. Table 1 shows the activation energies for growth of different IMC layers. It is found that the addition of Ni reduces the activation energy of Cu_6_Sn_5_ and total IMC, and improves the activation energy of Cu_3_Sn. However, when the Ni content is too high, loose IMC are likely to form at the interface of solder joints, leading to the reduction of solder joint strength and influencing the reliability of solder joints. Annealing the substrate is to heat the substrate to a temperature for a sufficient period of time and then cool. Annealing the substrate can change its microstructure, thereby inhibiting the germination and growth of IMC and improving the reliability of solder joints. Kim et al. [24] studied the influence of annealing Cu substrate on interfacial IMC and found that annealing Cu substrate can effectively inhibit the growth of IMC. The grain of the Cu substrate was refined during the annealing process, which made the structure more uniform and stable and eliminate the internal stress and tissue defects of the Cu substrate. That can effectively reduce the diffusion rate of Cu atoms, thereby suppressing the germination and growth of the interface IMC.

**Table 1.** The activation energy for growth of different intermetallic layers.

	Activation energy for the growth of (kJ/mol)			
Solder	Total IMC	Cu_6_Sn_5_	Cu_3_Sn	Reference
Sn3.8Ag0.7Cu	77.70	52.27	80.69	[25]
Sn3.8Ag0.7Cu-0.27 Ni	48.36	43.13	96.40	[25]
Sn3.5Ag0.5Cu	75.10	58.60	114.70	[26]
Sn3.5Ag	64.82	48.54	89.06	[27]
Sn0.5Ag4Cu	53–63	44–54	35–41	[28]
Sn3.5Ag0.5Cu	44.3	_	88.1	[29]

**Figure 3.**
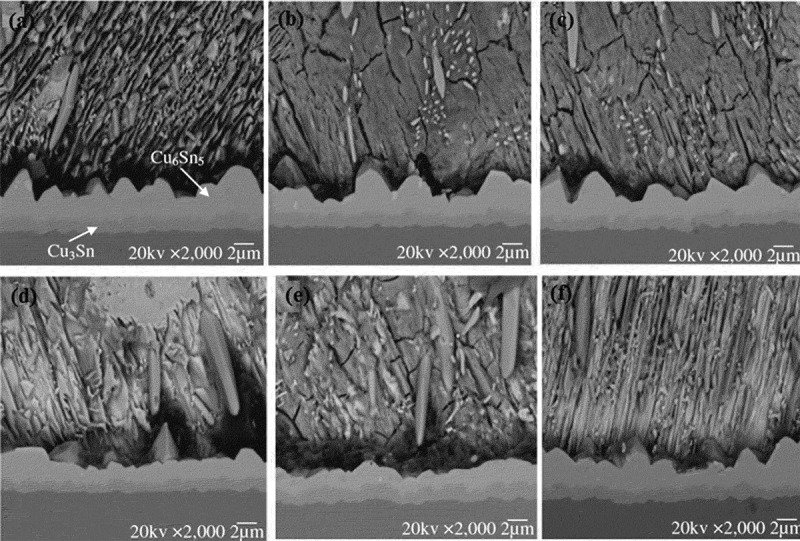
Cross-sectional SEM images of Sn3.0Ag0.5Cu-xTiO_2_ solder containing: (a) 0.0, (b) 0.02, (c) 0.05, (d) 0.1, (e) 0.3, and (f) 0.6 wt% aged at 190°C for 240 h [35], reproduced by permission from Tang et al., J Mater Sci. 2014;25(2):981–991.

The addition of alloying elements or metal particles to the lead-free solder can improve individual or overall properties of the solder [30], thereby reducing the effect of aging on solder joint reliability. Hu et al. [31] studied the effect of the addition of Ag on the interface reaction of SnBi/Cu solder and found that the addition of Ag can improve the reliability of SnBi solder joints. Mainly because SnBi solder joints are prone to Bi segregation during the aging process, and Bi segregation can cause brittleness of SnBi solder joints and reduce the reliability of solder joints. The addition of Ag can suppress the occurrence of Bi segregation and improve the reliability of solder joints. However, if the added Ag content is too high, a large amount of flaky Ag_3_Sn is formed in the solder, which affects the reliability of the solder joint [32]. Kanlayasiri et al. [33] investigated the effect of the addition of In on the reliability of low-silver SnAgCu solder joints during the aging process. They found that the thickness ratio of Cu_3_Sn and Cu_6_Sn_5_ layers decreased significantly with the increase of In content. The addition of In significantly inhibits the growth of the Cu_3_Sn IMC layer, thereby improving the reliability of the solder joint. Gain et al. [34] studied the effect of adding Al nanoparticles on SnAgCu lead-free solder. It was found that the addition of 3% Al nanoparticles can effectively improve the reliability of SnAgCu lead-free solder joints. Because the addition of the mass fraction of 3% of the Al nanoparticles reduces the thickness of the IMC layer on the cathode and anode interfaces of the solder joint, the brittleness of the IMC layer is greatly reduced, and the shear strength of the lead-free solder joint is enhanced. Tang et al. [35] studied the effect of the addition of TiO_2_ nanoparticles on the IMC growth of Sn3.0Ag0.5Cu solder joint interface in the isothermal aging process and found that the addition of TiO_2_ nanoparticles could effectively inhibit the germination and growth of IMC. The main reason is that the addition of TiO_2_ nanoparticles improves the activation energy of IMC and reduces the diffusion rate between Cu and Sn atoms, thus effectively inhibiting the growth of IMC. Figure 3 displays the cross-sectional SEM images of Sn3.0Ag0.5Cu-xTiO_2_ (x = 0.0, 0.02, 0.05, 0.1, 0.3 and 0.6 wt%) solder aged at 190°C for 240 h. It can be seen that the IMC layer thickness of Sn3.0Ag0.5Cu solder joint was significantly reduced after the addition of TiO_2_ nanoparticles. As the content of TiO_2_ nanoparticles increases to 0.1%, the total thickness of the IMC layer gradually decreases, as shown in Figure 3(a–d). However, when the content of TiO_2_ nanoparticles exceeds 0.1%, the total thickness of the IMC layer increases slightly, as shown in Figure 3(e,f).

### 2.2. Thermal cycling

During the service of electronic products, the periodic changes of the ambient temperature and the periodic switching of the circuit will cause the solder joint to be subjected to high and low-temperature cycles. Under the action of thermal cycling, the materials of the components and the substrate are thermally expanded. The linear expansion coefficient of the material was different, which resulted in the presence of alternating stress and strain of solder joint, thereby leading to the appearance of a large number of micro-cracks [36,37]. That can affect the reliability of solder joints. Therefore, it is necessary to study the effect of thermal cycling on solder joint reliability. The failure mechanism of lead-free solder joints under thermal cycling was studied by many experts and scholars [38,39], they observed that thermal fatigue cracks typically occur inside the bulk solder of joints near the interface regions.

For thermal cycling reliability of solder joints, there are many factors associated with the tests itself that can influence the interpretation of results. Zhou et al. [40] studied that the effect of continuous and interrupted accelerated thermal cycling on solder joint reliability. It was found that continuous thermal cycling led to solder joints with less recovery and much larger degree of recrystallization, whereas a strong and stable recovered microstructure formed during interrupted thermal cycling. Under the highly accelerated conditions, the networks of grain boundaries formed by recrystallization supplied favorable paths for cracks to propagate [41]. Hokka et al. [42] found that lower dwell temperature and lower dwell time, the ramp rate, temperature difference, dwell time, mean temperature have a significant impact on the thermal cycling reliability of solder.

**Figure 4.**
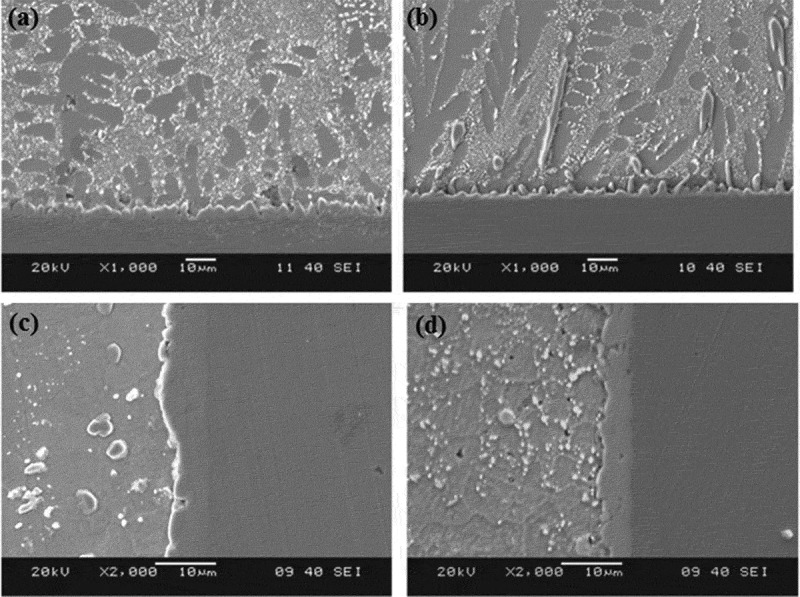
Interface microstructures: (a) SnAgCu/Cu after soldering; (b) SnAgCu-(nano-La_2_O_3_)/Cu after soldering; (c) SnAgCu/Cu after 1500 cycles; (d) SnAgCu-(nano-La_2_O_3_)/Cu after 1500 cycles [44], reproduced by permission.

Compared with the thermal shock test, the thermal cycle has a relatively long conversion speed, and the conversion speed is generally less than 20°C/min. At present, the temperature range mainly includes −55 ~ 125°C, −55 ~ 100°C, −40 ~ 125°C, −25 ~ 100°C, and 0 ~ 100°C. Li et al. [43] studied the IMC growth of SnAgCu/Cu solder joint interface under the thermal cycling conditions of −40 ~ 125°C and −25 ~ 125°C, and found that the thickness of IMC layer increased with the increase of thermal cycling times, and the growth rate of IMC interface under the thermal cycling conditions of −40 ~ 125°C was significantly higher than that under the thermal cycling conditions of −25 ~ 125°C. The main reason is that low temperature will cause cracks in IMC particles, which can be used as a diffusion channel to accelerate the growth rate of the IMC. Zhang and Gao [44] investigated the influence of the addition of La_2_O_3_ nanoparticles on the thermal reliability of SnAgCu solder joints under thermal cycling conditions of 55 ~ 125°C. It was found that the addition of La_2_O_3_ nanoparticles with the content of 0.1%wt significantly enhanced the thermal fatigue life of lead-free solder joints. Interface microstructures of SnAgCu/Cu solder are shown in Figure 4. It can be clearly seen that the addition of La_2_O_3_ nanoparticles transforms the morphology and thickness of Cu_6_Sn_5_ IMC at the SnAgCu/Cu interface, and the Cu_6_Sn_5_ gradually changes from scallop to stick-type. The thickness of the IMC is significantly reduced. As the thermal cycle time increases, the thickness of the IMC layer increases significantly. The main reason is that with the increase in the number of thermal cycle, the Sn atoms and Cu atoms in the interface layer will diffuse and react with each other, resulting in a significant increase in the thickness of the interface IMC layer. However, compared with the growth rate of IMC at the SnAgCu/Cu interface, the growth rate of IMC at the SnAgCu-(nano-La_2_O_3_)/Cu interface is significantly lower. Mainly because La_2_O_3_ nanoparticles effectively reduce the activation energy and diffusion coefficient of the IMC layer, thereby reducing the growth rate of IMC. Generally, the minimum standard for solder joint reliability of commercial electronic components is the lack of failure after 1000 cycles at 0 ~ 100°C, and the minimum standard for solder joint reliability of military electronic components is lack of failure after 500 cycles at −55 ~ 125°C [45].

**Figure 5.**
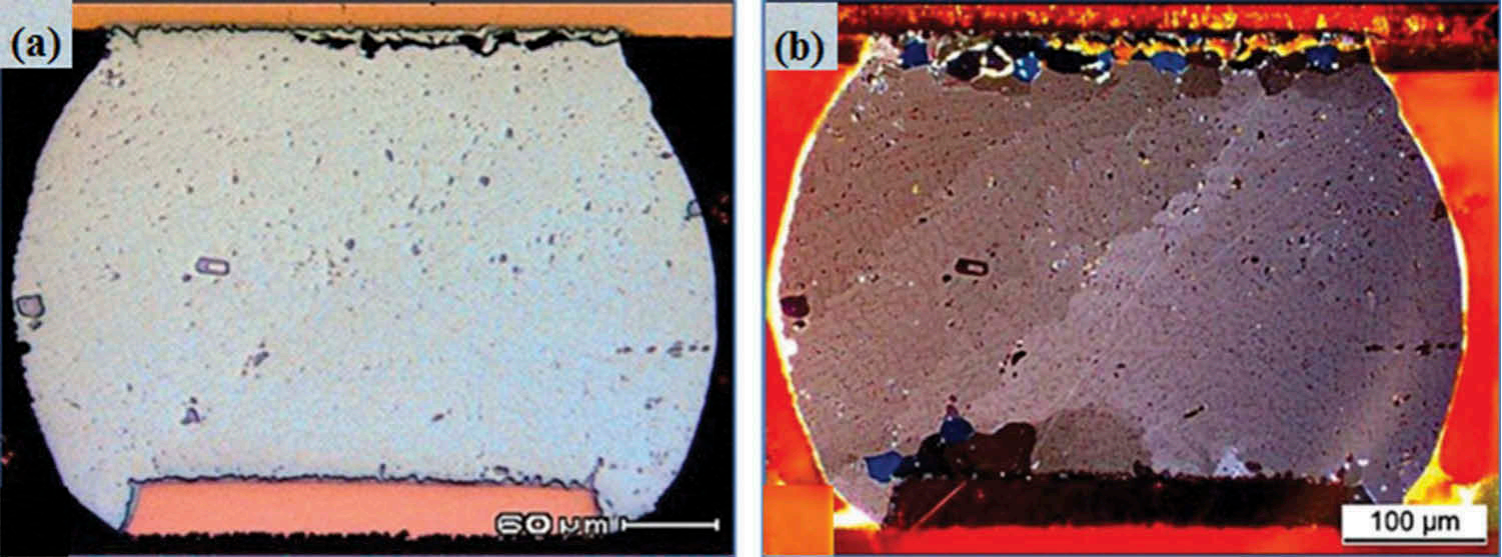
(a) Thermal fatigue crack through the bulk solder near the package pad side for Sn3.0Ag0.5Cu alloy and, (b) recrystallization in the bulk solder near the package pad side for Sn3.0Ag0.5Cu alloy [48], reproduced by permission from Shnawah et al., Microelectron Reliab. 2012;52(1):90–99.

Since the IMC during the thermal cycle usually exhibits brittleness, it tends to cause stress concentration in the bulk solder near the solder joints of the interface region, thereby causing the fatigue crack to germinate inside the bulk solder near the solder joint of the interface region [46]. Of course, the propagation of thermal fatigue cracks depends on the mechanical properties and microstructure of the bulk solder. Telang et al. [47] found that the thermal stress of the bulk alloy solder was unstable due to thermal stress during the thermal cycle test and Sn grain recrystallization. The typical ball grid array (BGA) solder joint failure [48] is shown in Figure 5. It can be seen that the bulk solder is broken, often accompanied by the appearance of recrystallization. The recrystallization will change the microstructure of the solder joint, thus reducing the thermal reliability of solder joints. High-silver SnAgCu solder can effectively reduce thermal stress due to its large amount of Ag_3_Sn and Cu_6_Sn_5_ in its structure, thus alleviating the recrystallization of solder in the thermal cycle test, so its thermal reliability is high [49]. However, the high-silver SnAgCu alloy solder has a high silver content, which greatly increases the cost of the solder. Sun et al. [50] investigated the effect of adding 0.1% Al nanoparticles on the mechanical properties of low-silver SnAgCu solder. It is found that the addition of Al nanoparticles significantly enhanced the mechanical properties of the solder joints, thus effectively improving the reliability of solder joints. Because the Al nanoparticles adsorbed on the grain boundaries of the interface, inhibited the germination and growth of the IMC layer, and reduced the grains size of the Cu_6_Sn_5_ IMC. Adding alloying elements such as Ni, Mn, and Bi to the low-silver SnAgCu alloy solder can also improve the thermal reliability of the solder joint [51].

Thermal fatigue life is an essential index to evaluate the thermal reliability of solder joints. Thermal fatigue test and finite element numerical simulation are mainly used to analyze the fatigue life of solder joints under thermal cycling. Early solder joint thermal cycle life was predicted from the thermal cycle life model based on the test. In the fatigue model based on energy, the fatigue life of solder joints is predicted by the strain energy in the stress-strain cycle [52]. A representative energy-based fatigue model was developed by Darveaux [53]. Shin et al. [54] studied the fatigue life of Plastic BGA (PBGA) lead-free interconnect solder joints using the Darveaux fatigue model. The fatigue point model based on plastic strain is mainly to predict the fatigue life of the solder joint by studying the number of failure cycles and the plastic stress of the solder joints in each cycle. There are three main models, namely Coffin-Manson solder joint fatigue model, Engelmaier solder joint fatigue model, and Solomon solder joint fatigue model. The Coffin-Manson solder joint fatigue model only considers plastic strain, Norris and Landzberg [55] modified it by frequency parameters, and the Engelmaier [56] solder joint fatigue model is mainly a correction for the Coffin-Manson solder joint fatigue model. In the fatigue strain model based on creep strain, the fatigue life of the solder joint is predicted mainly by the creep strain of the solder joint. There are two main models, the Syde model and the Knecht-Fox model. The Syde model [57] mainly considers grain boundary slip and matrix creep, while the Knecht-Fox model [58] and matrix creep strain amplitude relate the number of cycles when the solder joint is broken to the matrix creep strain amplitude. The fracture parameters are usually applied to analyze the elastoplastic fracture and fatigue life of engineering materials. The main model of fatigue model based on fracture parameters is the application of the intensity factor model [59]. However, these models do not consider fatigue and creep at the same time. Zhu et al. [60] proposed a new lead-free solder joint creep-fatigue life model at high strain rate, using the Monkman-Grant equation to evaluate creep damage and Coffin-Manson equation to evaluate fatigue damage.

**Figure 6.**
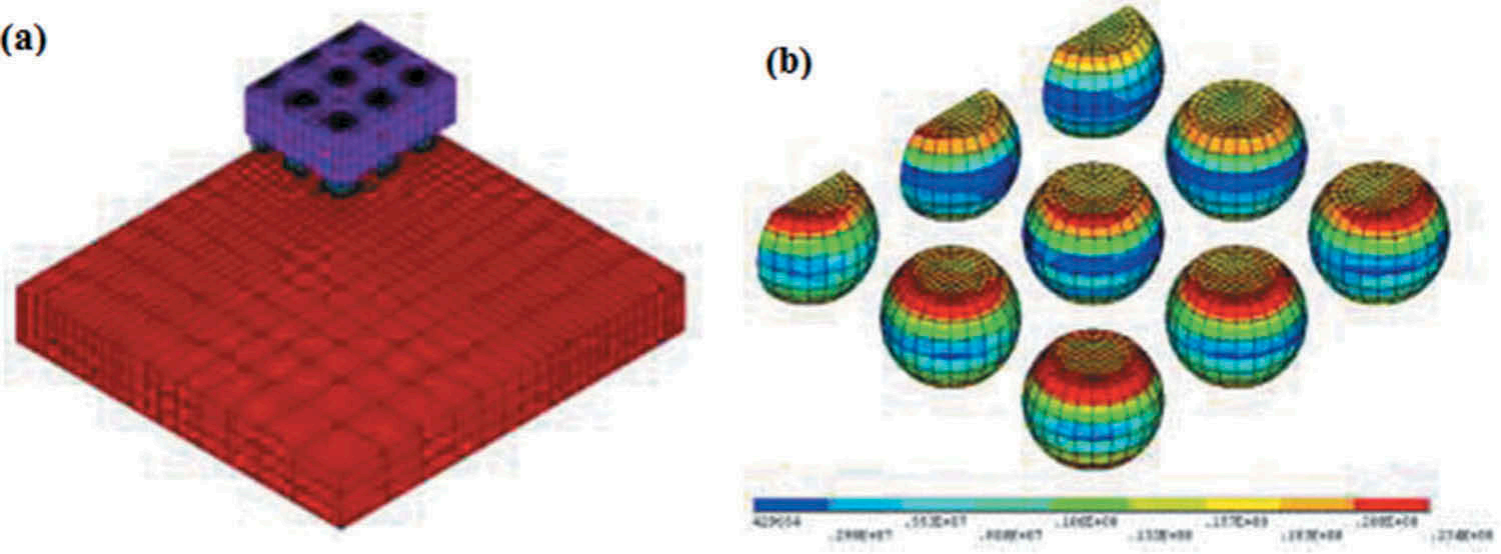
(a) Finite element model of WLCSP30 and (b) von Mises stress of solder joints [61], reproduced by permission from Zhang et al., Rare Metal Mater Eng. 2016;45(11):2823–2826.

However, with the development of electronic packaging technology to high function, high density, and high integration, the size and spacing of lead-free solder joints become smaller and smaller. Therefore, it is very difficult to predict the thermal fatigue life of solder joints using the test method. The emergence of finite element numerical simulation method provides an effective way to predict the thermal fatigue life of solder joints. The finite element method (FEM) is widely used to predict the fatigue life of solder joints. Zhang et al. [61] analyzed the stress-strain response of lead-free solder joints of wafer level chip scale packaging (WLCSP) devices under thermal cycling load and predicted the fatigue life of solder joints by FEM simulation. Figure 6 reveals the von Mises stress of SnAgCu solder and finite element mode of WLCSP device. It can be seen that the maximum stress of the lead-free solder joint of the device is concentrated at the upper surface of the corner of the solder joint array, from which the crack first originates, and then spreads and extends from the edge of the solder joint to the center along the interface. The addition of Fe particles can effectively inhibit the growth of the IMC and refine the microstructure of the solder, thereby improving the reliability of the solder joint. Chen et al. [62] studied the effect of IMC growth on the thermal reliability of solder joints by FEM and found that the growth of the IMC layer has an important influence on the thermal reliability of solder joints. A certain amount of IMC can enhance the strength of the solder joints, the IMC is dispersed in the lead-free solder inside the fine particles, which can play an *in* *situ* reinforcement similar to the composite material, thereby enhancing the thermal reliability of the solder joint. During the progress of the thermal cycle, the IMC is gradually thickened, and the stress concentration on the IMC interface is intensified, which leads to fracture damage at the IMC interface and reduce the thermal reliability of the solder joint [63]. Seunghyun and Youngbae [64] investigated the influence of embedded package solder joint materials on the fatigue life of solder joints under thermal cycling by FEM. It was found that the fatigue life of embedded solder joints increased and the fatigue life of Sn3.5Ag solder joints was higher. Mainly because of the low warpage of the embedded package, the creep strain of the solder joint and the total strain energy density are low, resulting in relatively high reliability of the embedded package solder joint. Le et al. [65] studied the influence of voids on the fatigue life of lead-free solder under thermal cycling by the finite element method. It was found that the fatigue life of lead-free solder joints depends not only on the size, position, and proportion of voids but also on the statistical distribution of voids. The effect of voids on the reliability of solder joints is significant. The main reason is that stress concentration tends to occur in the void area, which results in a significant weakening of the mechanical properties of BGA solder joints. The voids also significantly reduce the area of the joints of the solder joints, and the shear performance of the solder joints is significantly reduced, thereby reducing the reliability of the solder joints. However, the voids may have an effect on restraining the crack growth. Lau et al. [66] studied that the influence of voids on the solder joint, and found that the J-integral (driving force) for the crack without a void in front of it is a few times larger than that with a void in the solder joint. This means that the crack could be stopped by the void.

### 2.3. Thermal shock

During the service of electronic equipment, starting and shutting down will cause the high and low temperature of the internal solder joints to alternate. Due to the difference in thermal expansion coefficient between the solder joint and the component, the solder joint will withstand the cyclic shear stress during high- and low-temperature impact and will fail when the solder joint is subjected to a certain number of impacts [67]. The thermal reliability of the solder joint can be evaluated by a thermal shock test.

**Figure 7.**
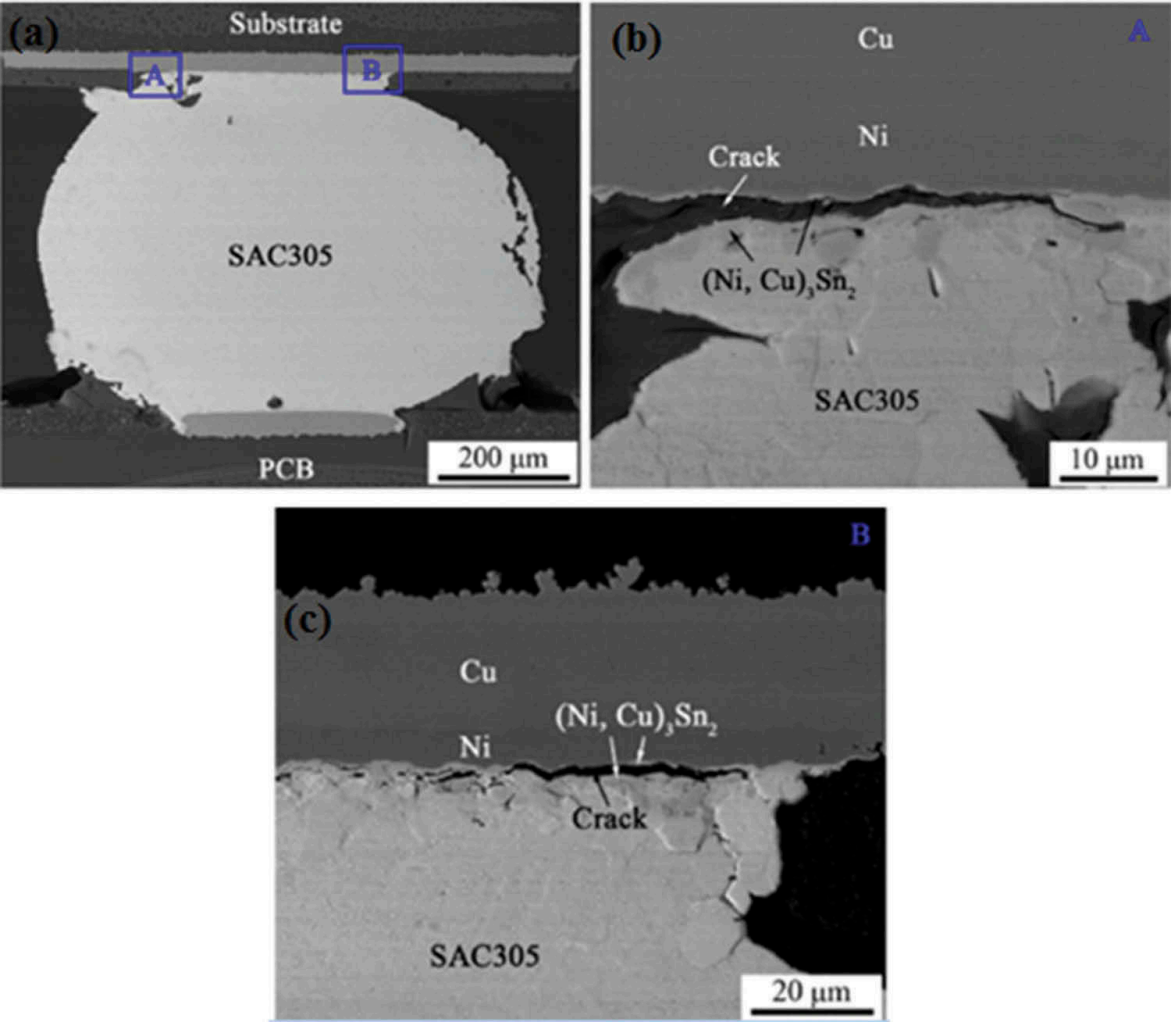
Cross-sectional SEM images of Sn3.0Ag0.5Cu solder after 300 cycles: (a) overall view of the solder, high magnification of (b) area A and (c) area B in (a) [71], reproduced by permission from Tian et al., J Alloys Compd. 2019;777:463–471.

As the number of thermal shocks increases, the IMC layer at the solder joint interface gradually becomes thicker. A thin and continuous IMC layer at the interface facilitates the formation of reliable solder joints [68]. The thicker interface IMC tends to make it exhibit brittle characteristics, which causes the shear strength of the solder joint to be significantly reduced [69], thereby reducing the reliability of the solder joint. Excessively thick IMC layers are also susceptible to stress, resulting in stress concentrations that can cause cracks in the solder joints, and ultimately lead to solder joint failure. Sharma et al. [70] studied the influence of thermal shock on the IMC growth of SnAgCu solder joints at −65 ~ 50°C and found that as the thermal shock period increases, the pores are dense due to the diffusion of atoms, and the Cu_6_Sn_5_ IMC is thin. The long scallops gradually thicken and transform into a flat interface with low surface energy. Tian et al. [71] studied the effect of thermal shock at 77 K to 423 K extreme temperature on the reliability of the PBGA Sn3.0Ag0.5Cu solder. It is found that the reliability of the solder joint after thermal shock decreased significantly. Figure 7 reveals the cross-sectional views of the solder after 300 cycles. It was found that cracks appeared in the (Ni,Cu)_3_Sn_2_ IMC layer both in the edge area and the neck area of the solder joint. Mainly because the (Ni,Cu)_6_Sn_5_ IMC at the interface is gradually converted into (Ni,Cu)_3_Sn_2_ IMC during thermal shock, while (Ni,Cu)_3_Sn_2_ IMC exhibits brittleness under the action of thermal stress [72]. It is easy to cause cracks, which affects the reliability of solder joints. Huang et al. [73] studied the structural evolution of Sn-6Bi-2Ag-0.5Cu solder joints during thermal shock and found that Cu-Sn IMC germinated and grew at the interface during thermal shock. Although the dispersed Ag_3_Sn in the solder joint is a relatively stable compound, it can increase the solder joint strength and improve the fatigue life of the solder joint [15]. However, the Ag_3_Sn phase and the Bi-phase are roughened during the aging process, thereby reducing the reliability of the solder joint. Tian et al. [74] studied the effect of thermal shock on the tensile strength of Sn3.0Ag0.5Cu solder in Quad Flat Package (QFP) and found that the tensile strength of solder joints decreased with the increase of thermal shock cycle. The reason is that as the thermal cycle increases, the IMC inside the lead-free solder gradually germinates and grows, and the matrix structure tends to coarsen, resulting in a decrease in the toughness of the Sn3.0Ag0.5Cu lead-free solder joint. The addition of trace amount of Zn to SnAgCu solder can reduce the mutual diffusion coefficient (D) of Sn and Cu, inhibit the growth of IMC, effectively reduce the growth rate of crack [75], and significantly enhance the thermal shock resistance of lead-free solder joints. Table 2 reveals the diffusion coefficients of different solder joint components.

**Table 2.** Diffusion coefficients of diverse solder joint components.

Solder	D/μm^2^ h^-1^	References
Sn0.3Ag0.7Cu/Cu	0.0105	[76]
Sn1.0Ag0.5Cu/Cu	0.0093	[76]
Sn3.0Ag0.5Cu/Cu	0.0092	[76]
Sn3.8Ag0.7Cu/Cu	0.0153	[63]
Sn3.8Ag0.7-Al/Cu	0.0107	[63]
Sn3.8Ag0.7-nono La_2_O_3_/Cu	0.0071	[44]

## 3. Vibration

The failure of the solder joint usually means that under the condition that the electronic component is in service, cracks appear in the stress concentration region of the solder joint due to external factors such as vibration and thermal cycling, and finally, the solder joint fails. Solder joint failure mainly consists of two aspects, namely high-cycle fatigue caused by vibration shock and low-cycle fatigue caused by temperature load [77]. Compared with low-cycle thermal fatigue, high-cycle vibration fatigue failure is more complicated. Therefore, the reliability of vibration has always been a key issue in the field of solder joint reliability research.

### 3.1. Vibration shock

According to statistics from the US Air Force, about 20% of electronic equipment failures are caused by vibration shocks [78]. When the electronic product is subjected to a vibration load, the PCB board undergoes large bending and deformation, and large alternating stress and strain are generated on the solder joint. The mechanical alternating stress between PCB board and solder joint will make the solder joint reciprocating tension and pressure, which may cause the generation, expansion and extension of cracks. In addition, the cracks may occur between chip and solder joint, which affects the reliability of electron devices.

**Figure 8.**
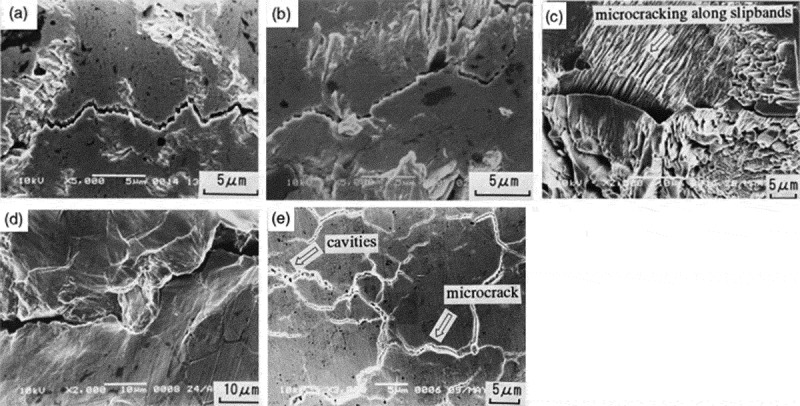
Fatigue crack paths in Sn3.5Ag solder: (a) R = 0.1, f = 10 Hz,; (b) R = 0.3, f = 10 Hz; (c) R = 0.5, f = 10 Hz; (d) R = 0.7, f =10 Hz; (e) R = 0.1, f = 0.1 Hz [85], reproduced by permission from Zhao et al., Int J Fatigue. 2001;23(8):723–731.

Many experts and scholars have studied the factors affecting the reliability of solder joint vibration. They found that different package modes and sizes, different solder joint materials and different solder joints have an underfill material that has a vital influence on solder joint vibration reliability [79,80]. Huang et al. [81] studied the effects of underfill arrays and non-underfill arrays on the vibration reliability of PBGA solder joints. It is found that the vibration fatigue life of PBGA solder joints in full fill array mode is higher than that of non-fully filled arrays. Mainly because the filling of lead-free solder joints with underfill can reduce the stress and strain in the solder joint under the random vibration load, thus effectively improving the vibration fatigue life of the solder joint. Song et al. [82] explored the effect of Cu content in SnAgCu solder on the vibration reliability of lead-free solder. It was found that Cu can effectively enhance the vibration resistance of lead-free solder and improve the vibration reliability of lead-free solder. Since the addition of Cu can enhance the dendrites of the Sn phase, and improve the toughness of the Cu_6_Sn_5_ IMC, thereby improving the vibration life of the solder joint. When the content of Cu in SnAgCu solder increases from 0% to 1.0%, the mechanical properties of lead-free solder joints are significantly enhanced, mainly because dendrites hinder the Sn-rich phase and reduce its damping, so that its anti-vibration performance is obviously enhanced. Barry et al. [83] investigated the difference in vibration reliability between Sn37Pb solder joints and Sn0.7Cu0.05Ni, Sn3.0Ag0.5Cu lead-free solder joints, and found that Sn37Pb solder joints have significantly higher vibration reliability than lead-free solder. Liu et al. [84] studied the vibration reliability of BGA package under random vibration load. It was found that the solder joints located at the four corners of the BGA package were the first to fail under random vibration load. The main reason is that the solder joints at the four most corners of the BGA package bear relatively large stress, which is likely to cause cracks and lead to the failure of the solder joints. As the vibration load applied to the PCB gradually increases, the fatigue life of the BGA solder joint decreases gradually. The stress on the solder joint gradually increases with the increase of the vibration load, thereby causing the solder joint to fail. Zhao et al. [85] studied the effect of vibration load on the fatigue fracture behavior of SnAg alloy and found that the magnitude and frequency of the load constrain the fatigue fracture behavior of SnAg alloy. With the increase of stress ratio and frequency, the fatigue crack growth rate increases gradually. The fatigue crack propagation behavior of the SnAg alloy transformed from cyclic dependence to time dependence, and the fracture mode changed from transgranular to inter-granular. Figure 8 shows the fatigue crack path of SnAg alloy under different stress ratios and frequencies. It can be seen from Figure 8(a) that when the stress ratio is 0.1 and the frequency is 10 Hz, the crack grows in a zigzag straight line in a transgranular manner. The fatigue crack path of the SnAg alloy tested at a stress ratio of 0.3 was very similar to the fatigue crack path with a stress ratio of 0.1, as shown in Figure 8(b). In the case of a high-stress ratio (R ≥ 0.5) and a low frequency (f = 10 Hz), a fatigue crack path is often observed on the surface of the sample as shown in Figure 8(c-e).

### 3.2. Drop

**Figure 9.**
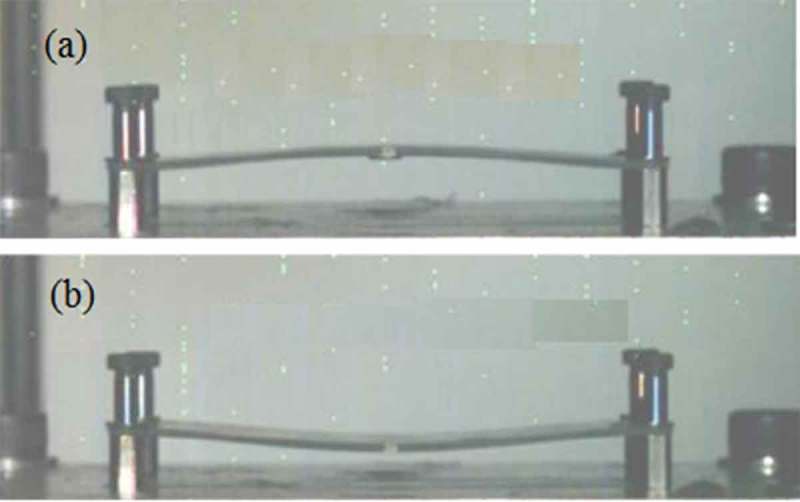
Bending diagram of PCB board: (a) bend upward, (b) bend down [86], reproduced by permission from Wu et al., Reliab. 2018;80:213–222.

With the development of electronic packaging technology toward high density, high functionality, and high integration, electronic components are gradually shifting to portable and miniaturized. In the process of using portable electronic products, they are often affected by drops and external impacts, causing the internal failure of the solder joints. As the impact of drop impact on the reliability of electronic products is increasingly prominent, solder joint failure is the primary cause of electronic product failure. Therefore, how to ensure the reliability of solder joint drop has become an essential research topic. The up and down bending of the PCB is the main reason for the failure of the solder joint during the drop process [86]. The up and down bending of the PCB board is shown in Figure 9.

Usually, the study of solder drop impact is divided into the product grade and board grade. Since the product-level drop test can only be carried out after the completion of the electronic product, which is unfavorable for the product development cycle and high in cost, the reliability of the electronic component is mainly studied through the board-level drop test. In July 2003, Joint Electron Device Engineering Council (JEDEC) released the test standard JESD22-B111 for board-level drop [87], which is widely used to test the drop reliability of portable electronic components. Wu et al. [88] were the first to study the drop reliability of portable electronic components through the board-level drop test. They established the drop test model of mobile phones through HYPERMESH and analyzed the failure conditions of mobile phones under the impact of dropping. Liu et al. [89] studied the reliability of BGA lead-free solder under plate-level drop impact and found that the solder joints of the four outermost corners of the BGA package failed first. The peeling stress is mainly caused by PCB bending vibration and mechanical shock, and PCB bending vibration is the main failure cause of solder joints under drop impact. The outermost corners are subjected to more stress, thereby resulting in the solder joints of the four outermost corners of the BGA package failed first.

**Figure 10.**
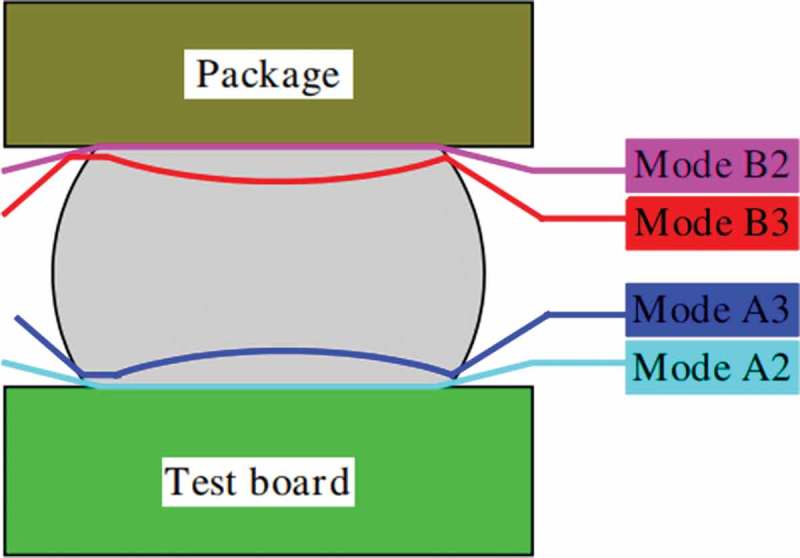
Schematic of failure modes [94], reproduced by permission from Yeh et al., Microelectron Reliab. 2006;46(7):1172–1182.

Qu et al. [90] also studied the reliability of BGA lead-free solder joints under board-level drop impacts and found that solder joint failures mainly occur at lower load levels of solder and PCB interfaces. Mainly because of a relatively lower impact level, there is a large strain energy density at the interface between the solder and the PCB, resulting in solder joint failure. There are two typical board-level drop tests, the free drop test [91] and the pulse-controlled drop test [92]. Yuan et al. [93] studied the reliability of solder joints in BGA packages by free fall test. It was found that impact energy was the main cause of solder joint cracks. The reciprocating bending deformation of PCB boards is the main cause of crack propagation and solder joint failure. However, these two methods do not accurately predict the drop reliability of solder joints under continuous drop conditions. Yeh et al. [94] proposed an excitation method combining implicit time integration and studied the transient structural response of a continuously dropping board-level package, found that there are four main solder joint failure modes during the drop test, as shown schematically in Figure 10. The description of these failure modes is shown in Table 3. The IMC layer is the main failure mode of the solder joint, mainly because the tensile and compressive amplitude is large, and it is mainly concentrated at the most corner of the peripheral solder joint. Luan et al. [95] established a dynamic monitoring system to monitor the entire failure process of solder joints by monitoring the voltage variation of the PCB through a DC power supply. The dynamic resistance method is suitable for all types of solder joint failure standards.

**Table 3.** Description of failure modes [94], reproduced by permission.

Modes	Characteristics	
A_2_	IMC fracturing	On test board side
A_3_	Solder fracturing	On test board side
B_2_	IMC fracturing	On package side
B_3_	Solder fracturing	On package side

**Table 4.** Failures caused by tin whiskers [106], reproduced by permission.

Product name that caused the accident	Analysis of the cause of the accident
F15 radar	Tin whisker growth on the tin plating layer leads to short circuit
Cardiac pacemaker	Tin whisker growth leads to short circuit
US missile incident	Tin whisker growth on Sn-plated transistors and relays, causing short circuits
Phoenix Air-to-Air Missile	Short circuit caused by tin whiskers
Patriot II missile	Tin whisker growth on tinned pins
Galaxy IV satellite	Tin whiskers grow on the Sn-plated relay, causing a short circuit, causing the satellite to lose control.
SOLIDARIDAD satellite	Tin whiskers grow on the plated Sn relay, causing a short circuit, causing the satellite to lose control.
Other satellites	Tin whisker growth disables the control processor
Nuclear device	Tin whisker growth on the Sn-plated layer of the relay
Rocket engine ignition	The appearance of tin whiskers shorts the housing and wires during assembly and testing.

**Figure 11.**
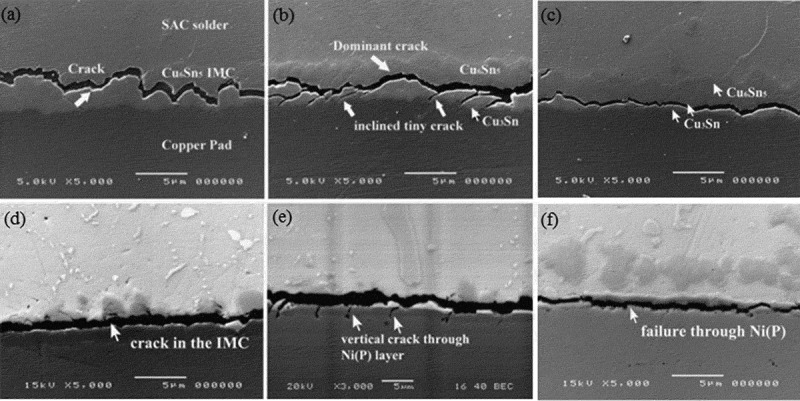
Drop impact crack path of test boards: (a) SAC/Cu-OSP, (b) SAC/Cu-OSP after 500 TC, (c) SAC/Cu-OSP after 1500 TC, (d) SAC/ENIG,(e) SAC/ENIG after 500 TC, (f) SAC/ENIG after 1500 TC [99], reproduced by permission from Xu et al., J Electron Mater. 2008;37(6):880–886.

Lai et al. [96] studied the drop reliability of thin fine-pitch BGA (TFBGA) lead-free solder joints by drop test and found that solder joints with lower Ag content can enhance the drop resistance of electronic products. Jee et al. [97] investigated the effect of Zn addition on the drop resistance of Sn3.5Ag lead-free solder and found that the addition of Zn alloy elements significantly enhanced the drop resistance of lead-free solder. Because the addition of Zn element refines the microstructure of Sn3.5Ag lead-free solder alloy, inhibiting the germination and growth of Ni_3_P, Ni_3_SnP, and Ni_3_Sn_4_ IMC. Mishiro et al. [98] studied the effect of underfill on the drop reliability of BGA/CSP packages and found that underfill can effectively improve the drop reliability of BGA/CSP packages. Mainly because underfill can reduce the stress strain of the BGA/CSP package during the drop impact, which enhances the drop reliability of the BGA/CSP package. Xu et al. [99] studied the effect of thermal cycling on the reliability of the solder joint drop and found that thermal cycling reduces the drop resistance of solder joints. Prior to thermal cycling, boards with Cu-OSP surface treatment had higher drop impact reliability than boards with ENIG boards. The result is reversed after thermal cycling, because the SAC/Cu-OSP after thermal cycling will cause the Kirkendall cavity at the interface between the IMC and the Cu substrate, thus affecting the drop impact reliability of the SAC/Cu-OSP. The crack paths of the SAC/Cu-OSP and SAC/ENIG test boards are shown in Figure 11. It can be seen from Figure 11(a–c) that the crack path of SAC/Cu-OSP is irregular, and as the thermal cycle time changes, the crack propagation path gradually changes from Cu_6_Sn_5_ IMC to Cu_3_Sn. As the thermal cycle time increases, the crack propagation path gradually changes from the interface IMC to the Ni(P) layer, as shown in Figure 11(d–f).

Although the reliability of lead-free solder joints under vibration and drop has been studied at home and abroad, the domestic understanding and research data on the reliability of vibration and drop of lead-free solder joints are very limited, which requires further research.

## 4. Tin whisker

**Figure 12.**
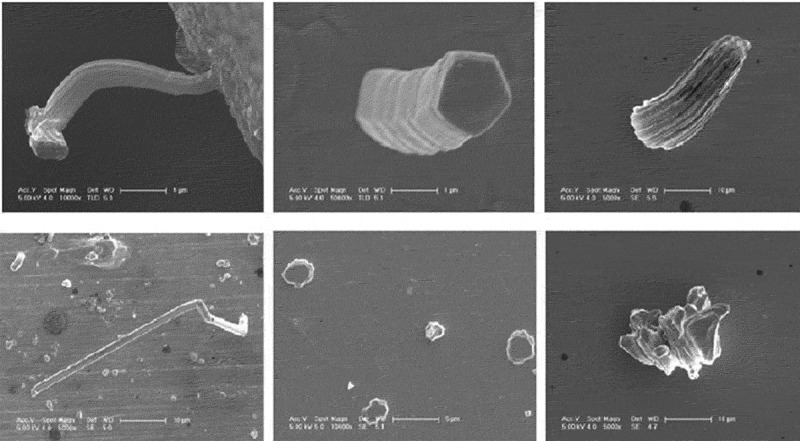
Tin whiskers of various shapes [102], reproduced by permission from Yu et al., Microelectron Reliab. 2010;50(8):1146–1151.

Tin metal is generally used as a connecting material and a plating material, but due to its lower melting point, whisker problems are easily generated. The diffusion of elements near room temperature is the basic driving force for the formation and growth of whiskers, while the diffusion rate of low-melting metal atoms is high, and whisker is prone to occur when pressure is applied slightly on the coating [100]. Tin whisker refers to the electronic device in the process of long-term storage and use, under the action of mechanical and temperature factors, the elements diffuse from the inside to the surface of the coating, will grow some whisker crystals on the surface of the high tin coating and grow. The main ingredient is tin [101]. Tin whiskers typically range in length from a few microns to tens of millimeters, with diameters ranging from 0.2 ~ 5 μm. They come in a variety of shapes [102], including needle-like, columnar, bifurcated, short columnar, tortuous, knurled, and bunched whiskers, as shown in Figure 12. At room temperature, the shape of tin whisker is mostly needle-like or silky; at high temperatures, the shape of tin whisker is mainly composed of stacked entangled flocks, mounds, and tumors.

### 4.1. Instances of whisker-induced problems

**Figure 13.**
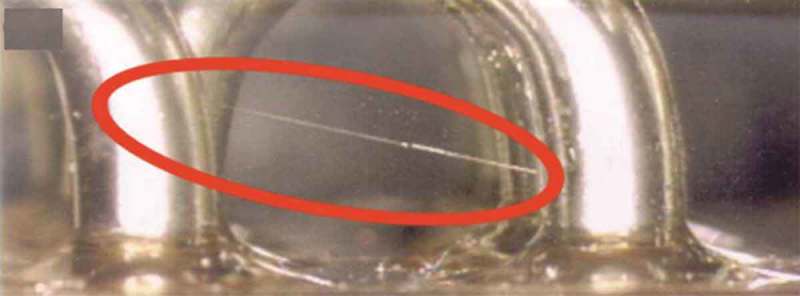
Electrical short circuits induced by whiskers [104], reproduced by permission from Zhang et al., J Mater Sci Technol. 2015;31(7):675–698.

**Figure 14.**
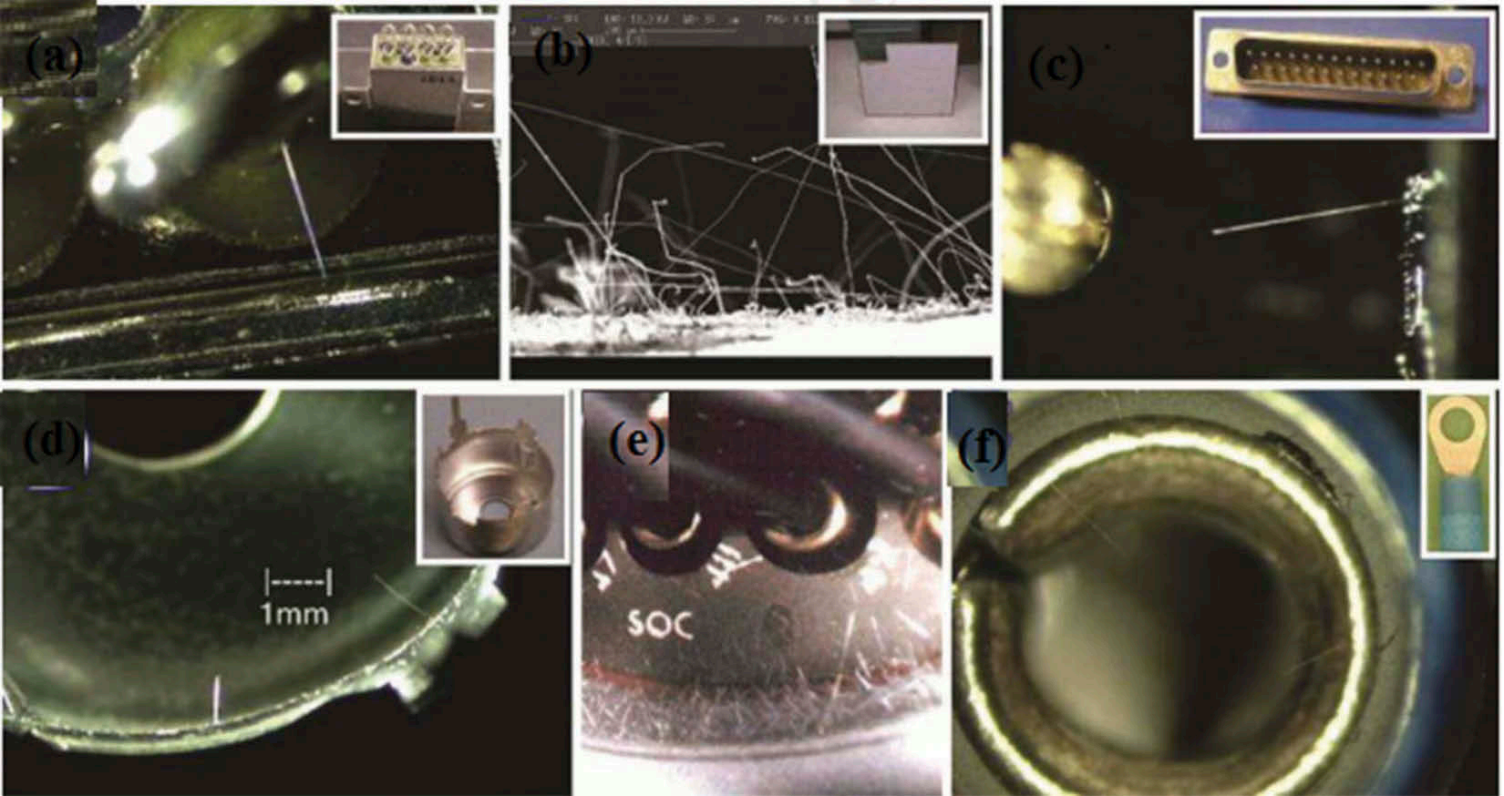
(a) Tin whiskers on relays, (b) zinc whiskers on the floor of computer room, (c) tin whiskers on D-sub connector, (d) tin whiskers on transformer can, (e) cadmium whiskers on connector, and (f) tin whiskers on lugs [104], reproduced by permission.

Tin whisker has a strong current load capacity, and its growth to a certain extent will connect two adjacent solder joints, causing short circuit of the electronic device [103]. The short circuit caused by the tin whisker is shown in Figure 13 [104]. When the applied current is less than 10 mA, the tin whisker will cause a permanent short circuit. When the applied current is greater than 10 mA, tin whisker can cause intermittent short circuit [105]. The tin whisker short circuit easily discharges the metal to form an electric arc, causing irreversible damage to the electronic product. Historically, countless failures caused by tin whiskers have had irreparable consequences. Figure 14 reveals several whisker cases on electrical components, for examples. The case of an accident caused by tin whiskers is shown in Table 4 [106]. Pb can hinder the grain boundary migration of Sn. Therefore, the electronic industry uses tin-lead alloy plating as a component pad plating to achieve the effect of relieving and suppressing whisker growth. However, with the lead-free process of electronic components, lead-free solders have gradually replaced SnPb solders. Further research is needed on the reliability of lead-free solder joints that must be caused by Sn.

### 4.2. The growth mechanism of tin whiskers

**Figure 15.**
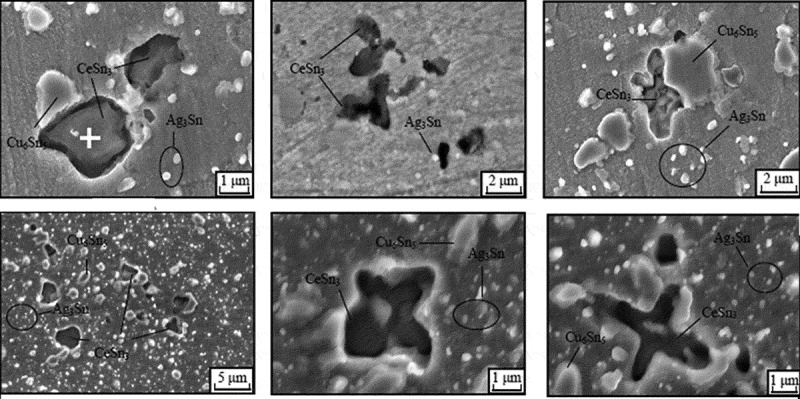
Rare earth phase inside SnAgCuCe solder joint [117], reproduced by permission.

In 1951, Compton et al. of Bell Labs discovered that metal whiskers germinated and grew on the surface of Sn, Al, Zn and other coatings [107]. The mechanism of whisker growth was studied by many experts and Tu believed that residual stress in solder joint component leads or coatings provided the driving force for whisker growth [108]. Zhang et al. [109] studied the main source of whisker growth and found that compressive stress is the driving force for whisker growth. Because the Cu atoms in the vicinity of the interface diffuse and dissolve and form the IMC over time, and the IMC generates compressive stress by growing them inside of the plating. The compressive stress in the coating is not only derived from the internal compressive stress of the coating caused by IMC germination and growth, but also from the residual compressive stress inside the coating caused by macroscopic stress, the internal compressive stress of the coating caused by surface moisture erosion, and the thermal expansion of the coating and the base metal. The thermal stress inside the coating caused by the coefficient mismatch. The recrystallization mechanism of tin whisker growth was proposed by Vance [110], who believed that the plated structure after plating required a recrystallization process to transform into a structure suitable for whisker growth. Noriyuki et al. [111] found that compressive stress causes recrystallization of tin in the coating, which causes whisker growth, and stable grain boundaries promote whisker growth. Nevertheless, Jagtap et al. [112] found that the structure of grain boundary basically keeps unchanged and whiskers grew from pre-existing grains, so they thought that the recrystallization mechanism of tin whisker growth was unreliable and grain boundary diffusion can be the dominant mass transport mechanism for the growth Sn whisker. The oxidative rupture mechanism was proposed by Kariya et al. [113], who believed that the internal stress was released by the defective portion of the plating oxide film, resulting in the growth of whiskers. But Sn whisker can grow in ultra-high vacuum, which means that oxidation is not the main driving force of whisker growth [114]. Chakraborty and Eisenlohr [115] found that whisker could continue to grow as long as the hydrostatic stress in the collection volume surpasses the energy needed to produce a fresh whisker surface. Hence, stress is the most commonly accepted driving force for the growth of Sn whisker. Adding the proper amount of rare earth elements can effectively improve the wettability and mechanical properties of lead-free solder joints, but the addition of excess rare earth can easily cause the growth of tin whiskers on the rare earth phase [116]. Zhang et al. [117] studied the effect of rare earth element Ce on the internal structure of lead-free solder joints. It was found that various shapes of CeSn_3_ phase appeared inside the solder joint, as shown in Figure 15. It can be seen that the size of the CeSn_3_ particles is significantly larger, followed by Cu_6_Sn_5_ and Ag_3_Sn. A large amount of Ag_3_Sn nanoparticles are distributed inside the solder joints, which significantly improves the mechanical properties of the solder joints, thereby greatly enhancing the reliability of the solder joints. However, when the added rare earth element is excessive, the growth of whiskers is also induced. When the Ce content added is less than 0.1%, the rate of whisker growth is remarkably lowered. However, when an excessive amount of Ce is added, tin whisker grows on the surface of the rare earth phase CeSn_3_. The main reason is that oxygen continues to diffuse into the intermetallic compound and react with the rare earth. The volume of the CeSn_3_ metal compound after oxidation is continuously increased, resulting in a large compressive stress inside the compound. Tin atoms are subjected to compressive stress and are constantly extruded from the surface of the oxide, resulting in the formation and growth of filamentous whiskers [118].

### 4.3. The inhibition of tin whiskers

**Figure 16.**
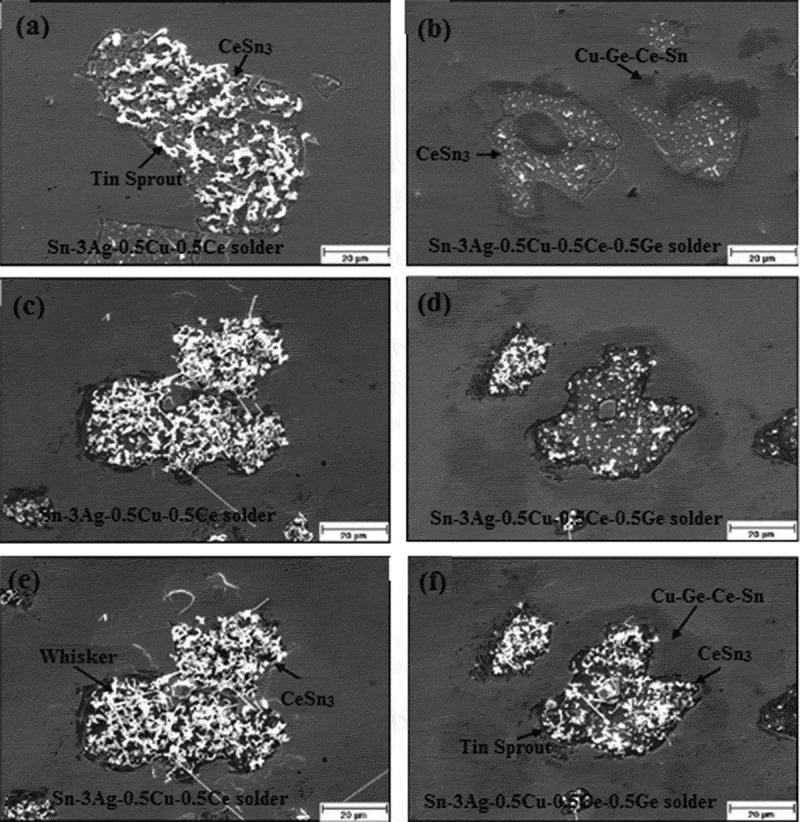
Tin whisker growth in Sn-3Ag-0.5Cu-0.5Ce-0.5Ge and Sn-3Ag-0.5Cu-0.5Ce-0.5Ge solders after air exposure at room temperature: (a) 12 h, (b) 12 h, (c) 170 h, (d) 170 h, (e) 674 h, (f) 674 h [122], reproduced by permission from Chung et al., J Alloys Compd. 2009;480(2):974–980.

The tin whisker not only severely restricts the application of rare earth-containing lead-free solders, but also has a great impact on the reliability of electronic devices. Therefore, the industry’s most concern is how to suppress the growth of Sn whiskers as much as possible. At present, experts and scholars deal with tin whisker by means of plating alloying, intermediate plating and heat treatment of surface coating. Howard et al. [119] studied the effect of pure tin matrix coating on whisker growth and found that the thinner the coating thickness, the smaller the growth density of tin whiskers. This is mainly because the thinner the thickness of the pure tin-tin plating layer, the closer it is to the diameter of the tin crystal grain, and the stress on the surface of the tin plating layer is not hindered and completely released. The alloying of the coating can effectively alleviate the growth of tin whisker. The alloying of the coating means that the finally formed coating is not pure Sn, but contains metal elements such as Ag, Pb, Fe, Bi, and Cu. Fisher et al. [120] studied the effect of alloying elements and Sn co-plating on whisker growth. The results show that the formation and growth of whiskers can be effectively alleviated by microalloying. Since microalloying can significantly reduce the driving force of whisker growth, thereby effectively suppressing the formation and growth of whiskers. However, the addition of Cu can promote the formation and growth of tin whiskers [121]. Because Sn and Cu will form IMC Cu_6_Sn_5_, which will lead to local stress generation and provide a driving force for tin whisker formation and growth. Chuang and Chi [122] studied the effect of 0.5% Ge on the growth of tin whiskers on the surface of Sn-3Ag-0.5Cu-0.5Ce solder joint and found that 0.5% Ge inhibited the growth of tin whiskers on the surface of lead-free solder joint. Because of the difficulty in the oxidation of CeSn_3_ intermetallic compounds after the addition of Ge, resulting in the lack of conditions required for the growth of tin whiskers. Figure 16 displays the whisker growth on the surfaces of Sn-3Ag-0.5Cu-0.5Ce and Sn-3Ag-0.5Cu-0.5Ce-0.5Ge solder exposed to air at room temperature. After 12 h of exposure at room temperature, the surface of Sn-3Ag-0.5Cu-0.5Ce solder has tin sprout growing, as shown in Figure 16(a). After 674 h of exposure at room temperature, the surface of Sn-3Ag-0.5Cu-0.5Ce solder has a large number of tin whiskers on the surface, as can be observed from Figure 16(e). Even when exposed to air for 674 h, only shorter tin sprouts formed on the surface of Sn-3Ag-0.5Cu-0.5C-0.5Ge solder, as shown in Figure 16(f). The intermediate plating layer can also suppress the growth of Sn whiskers. For the Sn surface plating layer, the material of the intermediate plating layer is generally Cu and Ni. The intermediate plating layer has three main functions, including the ability to effectively change the stress state inside the surface coating layer, and can significantly improve the corrosion resistance of the surface plating layer, and can serve as a diffusion barrier between the surface plating material and the substrate material. Britton [123] explored the effect of Ni intermediate coating on tin whiskers and found that Ni intermediate coating can significantly alleviate the growth of whiskers. Since the Ni intermediate coating can effectively eliminate the stress inside the film layer, eliminating the driving force of whisker formation and growth. Sudagar et al. [124] studied the effect of electroplating nickel on sulfamate and nickel on a watt bath as an intermediate layer on whisker growth. It is found that the nickel sulfamate-electroplated nickel and the watt liquid-electroplated nickel as the intermediate layer can inhibit the growth of tin whiskers on the electroplated surface, and the effect of the nickel plating on the silicon plating to suppress tin whisker is not as good as that of the sulfamate-electroplated nickel layer. This is mainly because the residual stress in the nickel plating layer of the Watt liquid is larger than that of the residual stress in the sulfamate-electroplated nickel layer, and the residual stress provides a driving force for the whisker growth. The heat treatment of the surface coating is a way to suppress the growth of whiskers, which are classified into annealing, melting, and reflux [125]. Melting and reflow are both melting and solidifying, except that the melting is immersed in oil having a temperature slightly above the melting point of the coating material. Annealing is the most common method and it is a metal heat treatment process, where the metal is slowly heated and then cooled at a suitable rate. Na et al. [126] studied the effect of heat treatment of surface coating on the whisker growth and found that heat treatment or reflow treatment at 150°C can effectively inhibit the growth of tin whisker at room temperature. They found that heat treatment effectively inhibited whisker growth. Barbara et al. [127] studied the effect of annealing on whisker growth. As a result, it was found that the sample after annealing was not stored and grown at room temperature. Samples that were not annealed were tested for a large amount of whisker growth when stored at room temperature for 2 months. This is mainly because the microstructure after heat treatment is changed, the internal stress is released, and the growth of tin whiskers can be effectively alleviated. However, heat treatment and Ni intermediate plating may not effectively suppress whisker growth due to oxidative corrosion [128]. The flux used in the package may effectively suppress whisker growth caused by oxidative corrosion because the residue left by the flux can cover the surface and thus function as a protective layer. However, flux residues can cause corrosion.

In recent years, a mass of research has been carried out on tin whiskers, and some achievements have been made. With the advent of the lead-free era, tin whiskers need more in-depth research: (1) In-depth study of the factors affecting the growth of tin whiskers and further explore the growth mechanism of Sn whiskers. (2) Exploring a highly adaptable tin whisker suppression method that can solve the package failure problem caused by whisker growth, thereby improving the reliability of the lead-free interconnect solder joint of the electronic package.

## 5. Electromigration

With the development of miniaturization and multifunction of electronic products, the density of solder joint on chip increases gradually. However, the spacing and size of solder joints decrease gradually, which leads to the increase of current density in solder joints [129]. The problem of electromigration between solder joints was becoming more and more serious, which has become a key factor affecting the development of high-density interconnection packaging technology. Therefore, the electromigration of lead-free interconnect solder joints in electronic packaging should be further studied and discussed.

**Figure 17.**
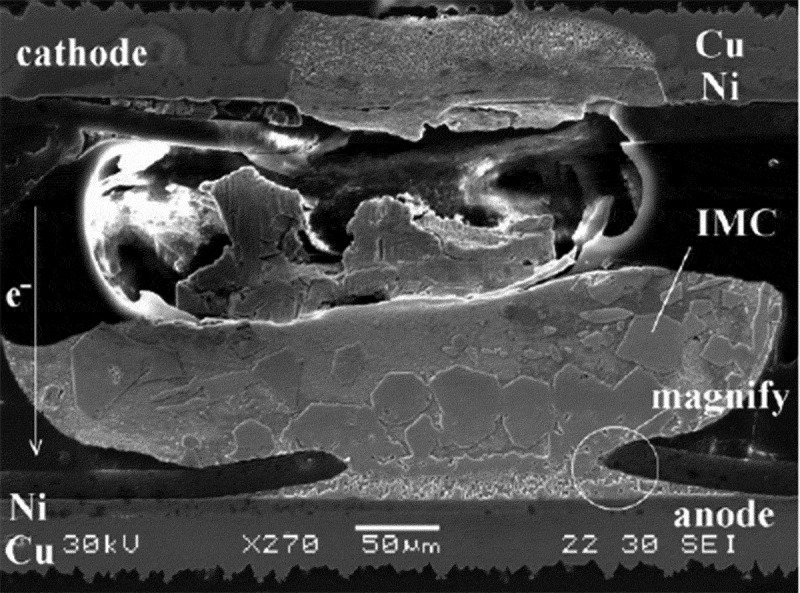
Electromigration failure of interconnected solder joints [130], reproduced by permission from Chang et al., Sci Rep. 2017;7(1):17950–17966.

Electromigration usually refers to the phenomenon that the structure of materials such as cracks and cavities is caused by the composition segregation of ions or atoms along with electron migration under the action of current in the interconnection metal or solder joint [130]. The electromigration failure of a typical interconnected solder joint is shown in Figure 17. The initial electromigration was driven by ‘electronic wind’, but electromigration failure is not an isolated phenomenon, and it is often accompanied by processes such as thermal migration and stress migration during electromigration. When an electric current passes through, the electrons form a strong flow field and the atoms are moved by its action [131]. When a large number of atoms are under the action of current, they will migrate from the cathode of the solder joint to the anode. It causes lattice pressure stress at the anode of the solder joint and induces the occurrence of extrusion and whiskers, etc. It also causes the crystal of the cathode of the solder joint to generate the gradual stress, which leads to the appearance of voids, and the formation of voids increases the diffusion channels and vacancies. The nucleation position causes the cavity to grow further and penetrate the entire solder joint. Wang et al. [132] studied the effect of temperature gradients in the solder joint on the cavity. It was found that the temperature gradient could change the diffusion coefficient and the resistivity of the voids in the solder joint, and the electromigration failure was intensified. The hot spots are easily formed at the voids, and the occurrence and growth of the solder joint hollow holes generally lead to the destruction of the outer lead type solder joints, which greatly affects the reliability of the solder joints. Huntington et al. proposed a formula for the effect of electronic wind on diffusion atoms [133].

(4–1) $$F_{em}=Z^{*}eE$$

where Z* is the effective charge number of electromigration, *e* is the charge carried by the electron, and *E* is the electric field strength.

The factors affecting electromigration have been studied by many experts and scholars, who believed that current density, temperature and solder joint composition had a significant influence on electromigration [134]. As the current density increases, the current stress generated by the current gradually increases, and the rate of atomic diffusion increases, which promotes the occurrence of electromigration. Chen et al. [135] studied the relationship between electromigration and current density. Metal interconnects in chips can generally withstand current densities of 10^5^ A/cm^2^ and above [136], but the maximum current density that solder joints can withstand in electronic packages is merely close to 10^4^ A/cm^2^. Therefore, it is generally considered that the critical current density value of the solder joint in the electronic package is 10^4^ A/cm^2^. When the current density of the solder joint exceeds the critical current density, the severity of the electromigration increases as the current density increases [137]. Tu et al. [138] investigated the effect of current density on the electromigration failure mode of solder joints by electromigration-accelerated test. It is found that the severity of atomic migration determines the electromigration failure mode of solder joints. The larger current density causes atomic migration. The movement of the current-crowded area is accelerated.

The higher the temperature, the higher the atom’s migration rate and the higher the probability of electromigration [139]. Lin et al. [140] studied the effect of temperature on the electromigration behavior of flip-chip solder joints and found that the severity of electromigration was significantly aggravated with increasing temperature. This is mainly because the activity of the Cu atom is remarkably enhanced with an increase in temperature, and the rate of movement is increased, so that migration by the electron wind is more intense. In the process of electromigration, the convexes and cavities formed will increase the linear resistance of the interconnection line, thus leading to the generation of joule heat [141]. The Joule heating effect causes a relatively large temperature gradient in the solder joint, which ultimately leads to thermal migration. When thermal migration is in the same direction as electromigration, thermal migration accelerates the process of electromigration. When the direction of thermal migration is dissimilar from the direction of electromigration, thermal migration will inhibit the occurrence of electromigration [142].

Solder joint composition has a significant impact on electromigration, and the easily diffused solder joint component accelerates the process of electromigration failure [143]. Chen and Huang [144] studied the effect of the addition of Ag on the electromigration resistance of SnBi solder joints. It was found that the electromigration resistance of solder joints gradually decreased with the decrease of Ag content. Because the Ag_3_Sn generated by the reaction with the Sn atom decreases as the Ag content of the solder joint decreases, and the Ag_3_Sn particles can hinder the migration of the atoms. Du et al. [145] investigated the effect of the addition of Sb on the electromigration resistance of lead-free solder joints. It is found that adding Sb element with a mass fraction of 0.1% would reduce the electromigration resistance of solder joints. The addition of the metal element Sb can react with the Sn atoms in the solder substrate to form a brittle phase SnSb compound, causing cracks under high current and destroying the matrix structure, thereby weakening the electromigration resistance of the lead-free solder joint. However, adding Sb with a mass fraction of 0.1% significantly enhances the mechanical properties of the solder [146], because the addition of Sb can effectively inhibit the germination and growth of intermetallic compounds. The addition of the alloying element Sb also enhances the creep resistance of the solder joint, because the addition of the Sb element significantly reduces the subcooling of the solder joint, thereby lowering the melting point of the solder and refining the microstructure [147], effectively improving the reliability of solder joints.

**Figure 18.**
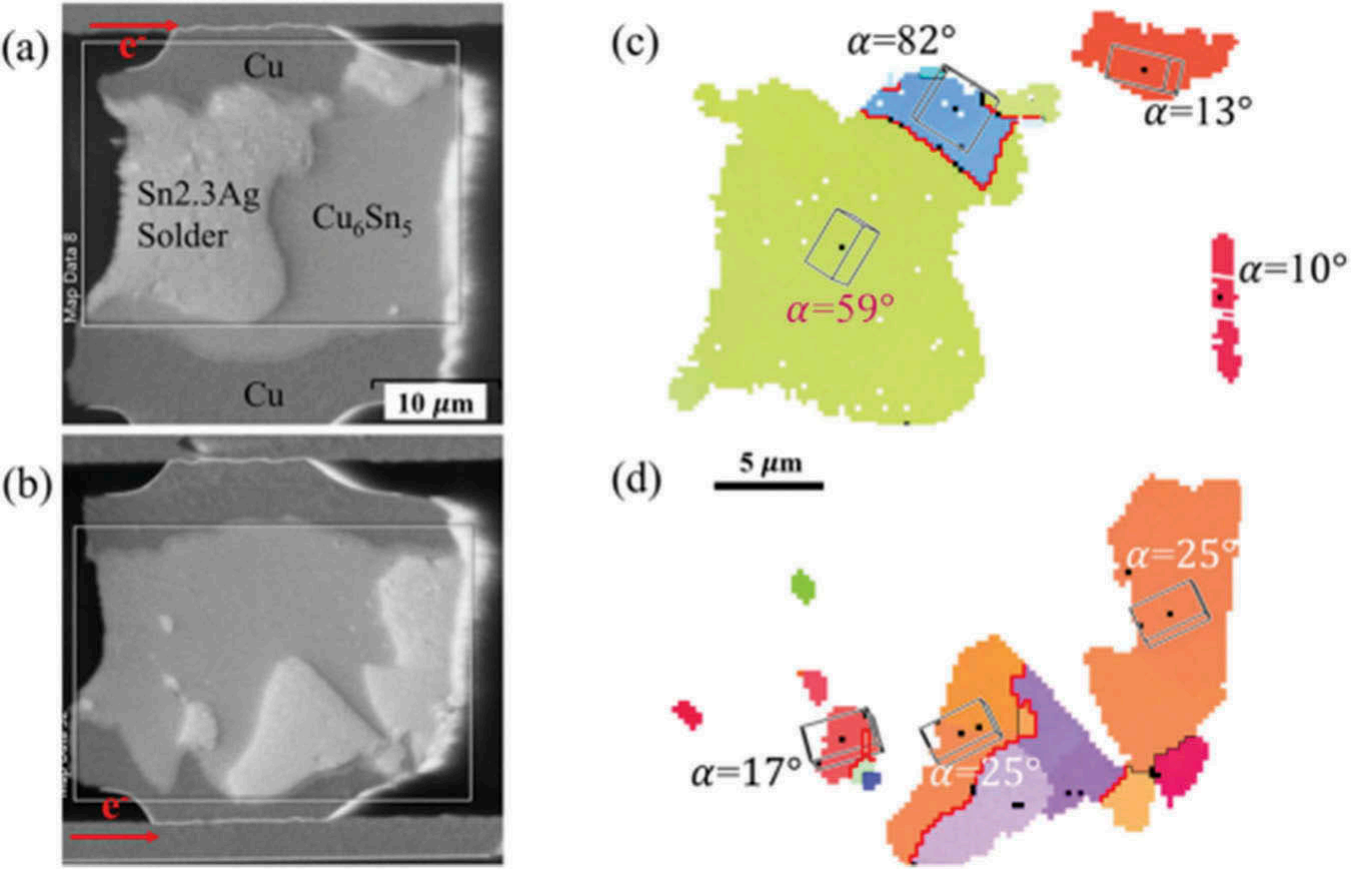
Rapid formation of Cu-Sn IMC in low-α-angle grains after electromigration: SEM images of the microbump (a) with a downward electron flow and (b) with an upward electron flow, (c) Corresponding OIM of the microbump in (a) and (d) corresponding OIM of the microbump in (b) [149], reproduced by permission from Shen et al., Scr Mater. 2017;128:6–9.

In addition, the microstructure of the solder joint has a significant effect on the electromigration. Lu et al. [148] found that when Ni atoms and Cu atoms diffuse rapidly along the c-axis of the Sn crystal grains, it is easy to cause a large amount of intermetallic compound dissolution at the cathode interface of the solder joint, thereby leading to the failure of electromigration at the microsolder joint. Shen et al. [149] studied the effect of Sn grain orientation on Cu-Sn intermetallic compounds formed during the electromigration of Sn2.3Ag solder joints. It was found that the growth of Cu-Sn intermetallic compounds during electromigration depends on the α angle (The c-axis of the Sn grain and the angle of the electron flow). The SEM images of the microbump are shown in Figure 18, and it can be seen that the grains with larger α angles have only less IMC formation. For Sn grains with an α angle of less than 25°, the growth rate of Cu-Sn intermetallic compounds during electromigration is faster, mainly because Cu atoms diffuse faster in the lower α-angle grains, thus accelerating the formation of Cu-Sn IMC during electromigration. Sn atoms usually exist in a solid tetragonal structure in the solid state, showing significant anisotropy in terms of thermal expansion coefficient and the diffusion coefficient [150]. Lee et al. [151] analyzed the influence of the direction of Sn grain diffusion on the electromigration of Sn3.0Ag0.5Cu lead-free solder joints and found that different Sn grain diffusion directions will produce different electromigration behaviors. When the current direction is perpendicular to the c-axis of the Sn grain, the electromigration behavior is weakened. When the current direction is parallel to the c-axis of the Sn crystal grains, Cu atoms diffuse along the c-axis gap of the Sn crystal grains, accelerating the occurrence of electromigration. Wang et al. [152] studied the effect of Sn grain structure on the electromigration reliability of Sn2.5Ag solder joints by backscattered electron diffraction method. It was found that Sn grain size and orientation contribute to the growth of intermetallic compounds and the formation of voids. Affects the electromigration reliability of solder joints. A single large-sized Sn grain easily leads to electromigration, mainly because the atomic diffusion coefficient largely depends on the size of the Sn grain. The larger the diffusion coefficient is, the faster the atomic diffusion is, which accelerates the occurrence of electromigration.

**Figure 19.**
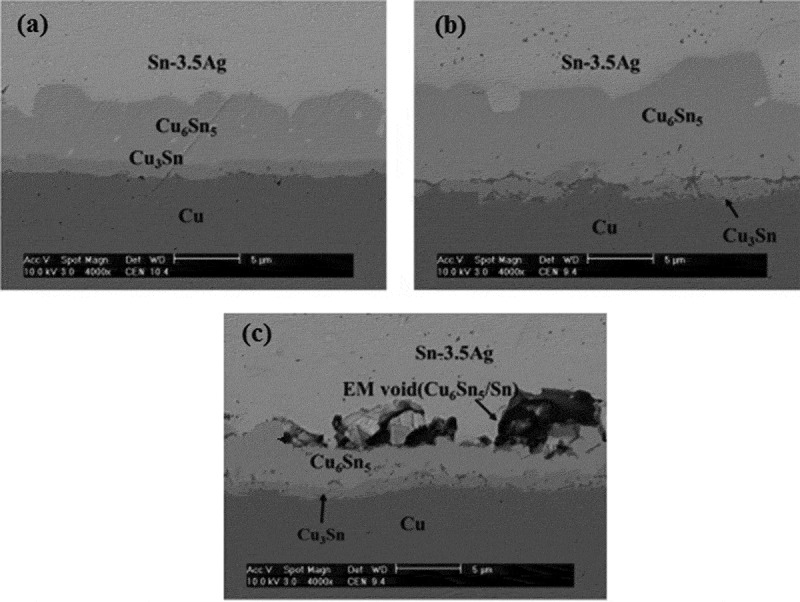
IMC morphology of solder joint interface before and after electromigration: (a) after brazing, (b) anode after electromigration, (c) cathode after electromigration [154], reproduced by permission from Jung et al., J Appl Phys. 2014;115(8):083708(10pp.).

Electromigration causes changes in the thickness and morphology of IMC at the solder joint interface, and intermetallic compounds are important for the reliability of solder joints. The brittle interface IMC may reduce the reliability of the solder joints, while the thin and continuous IMC can effectively improve the reliability of lead-free solder joints [153]. Jung and Yu [154] studied the growth of Cu_6_Sn_5_ IMC at the interface of Sn3.5Ag solder joints in the process of electromigration. It was found that the growth of Sn_5_ IMC at the interface of lead-free solder joints showed an obvious ‘polar effect’ in the process of electromigration. The interfacial IMC after electromigration is shown in Figure 19. It can be seen that the anode IMC of solder joints after electromigration significantly thickened, and promoted the growth of anode IMC. However, the thickness of cathode IMC at the solder joint became thinner, which indicated that electromigration inhibited the growth of cathode IMC and promoted the dissolution of cathode IMC. Voids appeared at the cathode interface after electromigration, mainly because Sn atoms diffused rapidly in places with high current density, and vacancies would be generated in the lattice, and further aggregation of vacancies would lead to the generation and growth of voids [155]. The stress concentration frequently occurs in the void area, which affects the reliability of solder joints. However, the ‘polar effect’ exhibited by the IMC growth at the solder joint interface is not absolute. Zhang et al. [156] studied the growth of IMC during solid-state electromigration and found that the IMC at the cathode interface of Cu/SnZn/Cu solder joints are larger than the thickness of the anode interface IMC, showing a significant ‘reverse polarity effect’. Huang et al. [157] studied the growth of IMC in Ni/SnZn/Ni solder joints during liquid-solid electromigration. It was found that the growth of IMC at the solder joint interface also showed a ‘reverse polarity effect’. Because the Sn atom preferentially diffuses to the anode of the solder joint, and the Zn atom is subjected to back stress, thereby diffusing toward the cathode of the SnZn solder joint, suppressing the dissolution of the cathode of the solder joint, and finally causing the cathode IMC of the solder joint to be higher than the anode.

**Figure 20.**
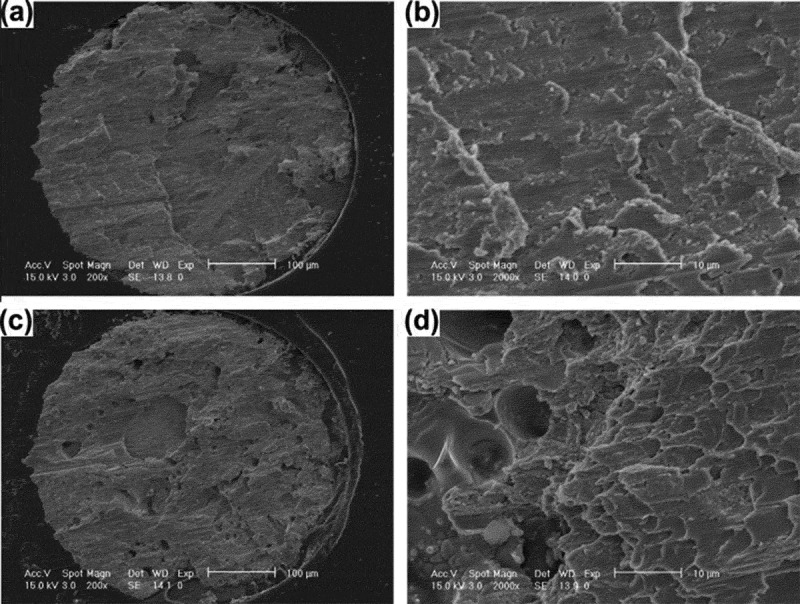
SEM fracture surfaces after ball shear test: (a) Sn-8Zn-3Bi solder, (b) Partial enlargement picture of (a), (c) 0.1 wt.% doped solder, (d) Partial enlargement picture of (c) [159], reproduced by permission from Hu et al., J Alloys Compd. 2013;580:162–171.

Electromigration can cause microscopic defects such as tiny voids inside the solder joint, resulting in a decrease in the mechanical properties of the solder joint. Zhang et al. [158] explored the effect of electromigration on the mechanical properties of Sn3.5Ag0.7Cu solder joints. The results show that electromigration will greatly reduce the tensile strength of lead-free solder joints. Mainly because electromigration causes structural defects such as voids in the cathode of the solder joint. The number of these structural defects will gradually increase with the extension of electromigration time, resulting in stress concentration, which will eventually lead to a significant decrease in the tensile strength of low-silver lead-free solder joints. Hu et al. [159] studied the effect of electromigration on the shear strength of Sn-8Zn-3Bi lead-free solder joints and found that the shear strength of solder joints after electromigration was significantly reduced. Because the IMC Cu_5_Zn_8_ formed by the reaction of Zn atoms and Cu atoms will gradually increase. The IMC is a brittle phase, which causes the plastic deformation ability of the solder joint to gradually weaken, and the fracture mode changes to the brittle mode [160], which ultimately reduces the reliability of the solder joint. The doping of 0.1% of graphene into the Sn-8Zn-3Bi solder significantly enhances the shear strength of the lead-free solder joint. Figure 20 displays the SEM fracture surfaces of the solder joint after the ball shear test. It can be seen from Figure 20(a,b) that the fracture surface of the Sn-8Zn-3Bi solder is relatively smooth, almost no dimple exists, and exhibits a remarkable brittle fracture mode. The fracture surface of the Sn-8Zn-3Bi solder doped with 0.1% graphene has a large number of dimples and exhibits a significant plastic fracture mode, as shown in Figure 20(c,d). Wang et al. [161] analyzed the mechanical properties of lead-free interconnected solder joints during electromigration through electromigration experiments. The results show that electromigration greatly reduces the shear performance of Sn3.8Ag0.7Cu lead-free solder joints. Since the lead-free solder joint interface, IMC will gradually increase with the extension of electromigration time, and the thicker interface IMC tends to make it exhibit brittle characteristics, which causes the shear strength of the solder joint to be significantly reduced. The addition of rare earth element Er can improve the shear properties of the solder alloy, because the rare earth elements are relatively active, which can refine the grain size of IMC and effectively inhibit the germination and growth of interfacial intermetallic compounds [162]. However, it does not cause changes in the bonding method and the chemical composition of the solder joint solder [163].

**Figure 21.**
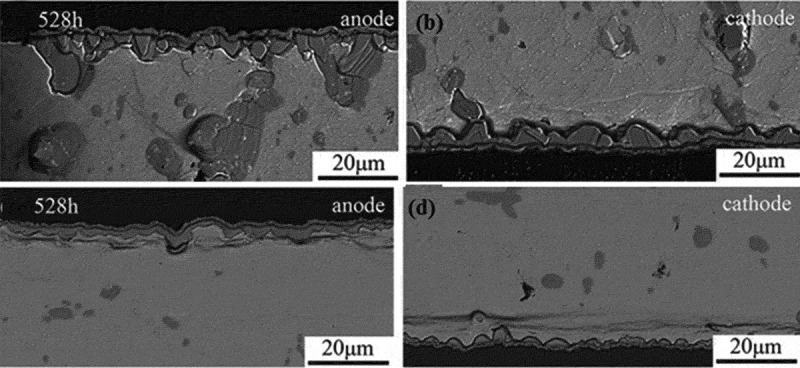
Microstructures of Sn3.5Ag-xCu solder joint after current stressing for 528 h: (a) 0, (b) 2% [167], reproduced by permission from Wang et al., J Electron Mater. 2016;45(12):6095–6101.

Preventive measures for electromigration have always been a vital issue in the electronics field. At present, the ability of solder joints to resist electromigration failure is improved mainly by adding alloying elements and controlling current density. The addition of suitable alloying elements can effectively enhance the electromigration resistance of the solder joints, thereby improving the reliability of the solder joints. Ma et al. [164] studied the effect of adding Co particles on the electromigration of Sn3.0Ag0.5Cu solder. The addition of element Co significantly inhibited the germination and growth of intermetallic compounds, thus improving the reliability of solder joints. The addition of Co particles can effectively inhibit the germination and growth of acicular and flaky IMC Cu_6_Sn_5_ and refine its microstructure, thereby greatly improving the mechanical properties of lead-free solder joints. Sun et al. [165] explored the effects of Bi and Ni addition on the electromigration of SnAgCu solder joints. The results show that the addition of Bi and Ni in the solder joint of SnAgCu solder reduces the melting point of lead-free solder, which effectively improves the electromigration resistance of lead-free solder joints. This may be because the addition of Bi and Ni to the lead-free solder effectively inhibits the growth of the intermetallic compound while significantly reducing the grain size of the intermetallic compound. Ma et al. [166] explored the effect of adding trace amounts of Ni on SnAgCu solder joints on electromigration and found that when the added Ni content is 0.45%, the damage caused by point migration can be effectively reduced. Wang et al. [167] studied the effect of Cu addition on the electromigration of SnAg eutectic solder joints. It was found that Cu with a doping content of 2% can effectively alleviate the occurrence of electromigration and reduce the surface damage to composite solder joints. Figure 21 reveals the microstructure of Sn-3.5Ag-xCu solder joint after current stressing for 528 h. It can be seen that Cu with a doping content of 2% significantly reduces the growth rate of IMC at the anode interface of SnAg solder joints, alleviates the polarity effect during electromigration, and greatly enhances the electromigration resistance of lead-free solder joints. As the current density increases, the Joule heat gradually increases. A vital way to mitigate electromigration is to control the current density. Transforming the current density can be achieved by changing the magnitude of the current and the size of the solder joint. Liang et al. [168] studied the effects of current density and Joule heat on the electromigration of SnAg solder joints. It is found that current density and Joule heat caused electromigration failure, which reduced the reliability of lead-free interconnect solder joints. The current density is appropriately reduced, and the electromigration failure is alleviated. This may be because at a relatively high current density, the diffusion rate of Sn atoms is significantly lower than that of Ag atoms, and the migration of Sn atoms is blocked by Ag atoms, resulting in a sharp increase in the internal pressure of the Sn-rich phase to form bumps, thereby reducing solder joint reliability. Huang et al. [169] studied the influence of Sn grain diffusion on the electromigration of Sn3.0Ag0.5Cu lead-free solder joints. They found that different Sn grain diffusion directions produced different electromigration behaviors. When the current direction is parallel to the c-axis of the Sn crystal grains, Cu atoms diffuse along the c-axis gap of the Sn crystal grains, accelerating the occurrence of electromigration. By controlling the temperature and current density, the direction of Sn grain diffusion can be effectively suppressed, thereby alleviating the occurrence of electromigration.

As electronic packaging is gradually becoming more miniaturized, multi-functional, and dense, the problem of electromigration in interconnected solder joints will become more and more serious. Electromigration does not usually occur alone, but is accompanied by stress fields, temperature gradients, and thermal diffusion effects. Therefore, it is necessary to study electromigration in lead-free interconnect pads under the alternate coupling of multiple loads.

## 6. Conclusions

In recent years, a great deal of research has been carried out on the reliability of lead-free solder joints for electronic packaging, and certain results have been achieved. For the reliability of solder joints during the service of electronic components, there are several aspects to be further studied: (1) Study on the reliability of solder joint under the composite condition of vibration and thermal; (2) The factors affecting the growth and the growth mechanism of Sn whiskers should be deeply studied, so as to explore a highly adaptive method of tin whisker inhibition and improve the reliability of lead-free solder joints during the service of electronic components; (3) Electromigration is usually not a single existence, and will be accompanied by the impact of the stress field, temperature gradient and thermal diffusion. The electromigration problem of lead-free solder joint that is in a variety of load alternating coupling need to be deeply discussed and researched.
